# Genome-Wide DNA Methylation Maps in Follicular Lymphoma Cells Determined by Methylation-Enriched Bisulfite Sequencing

**DOI:** 10.1371/journal.pone.0013020

**Published:** 2010-09-29

**Authors:** Jeong-Hyeon Choi, Yajun Li, Juyuan Guo, Lirong Pei, Tibor A. Rauch, Robin S. Kramer, Simone L. Macmil, Graham B. Wiley, Lynda B. Bennett, Jennifer L. Schnabel, Kristen H. Taylor, Sun Kim, Dong Xu, Arun Sreekumar, Gerd P. Pfeifer, Bruce A. Roe, Charles W. Caldwell, Kapil N. Bhalla, Huidong Shi

**Affiliations:** 1 Center of Genomics and Bioinformatics, Indiana University, Bloomington, Indiana, United States of America; 2 Medical College of Georgia Cancer Center, Medical College of Georgia, Augusta, Georgia, United States of America; 3 Department of Pathology and Anatomical Sciences, University of Missouri, Columbia, Missouri, United States of America; 4 Division of Biology, City of Hope Beckman Research Institute, Duarte, California, United States of America; 5 Department of Computer Sciences, University of Missouri, Columbia, Missouri, United States of America; 6 Advanced Center for Genome Technology, University of Oklahoma, Norman, Oklahoma, United States of America; Duke-National University of Singapore Graduate Medical School, Singapore

## Abstract

**Background:**

Follicular lymphoma (FL) is a form of non-Hodgkin's lymphoma (NHL) that arises from germinal center (GC) B-cells. Despite the significant advances in immunotherapy, FL is still not curable. Beyond transcriptional profiling and genomics datasets, there currently is no epigenome-scale dataset or integrative biology approach that can adequately model this disease and therefore identify novel mechanisms and targets for successful prevention and treatment of FL.

**Methodology/Principal Findings:**

We performed methylation-enriched genome-wide bisulfite sequencing of FL cells and normal CD19^+^ B-cells using 454 sequencing technology. The methylated DNA fragments were enriched with methyl-binding proteins, treated with bisulfite, and sequenced using the Roche-454 GS FLX sequencer. The total number of bases covered in the human genome was 18.2 and 49.3 million including 726,003 and 1.3 million CpGs in FL and CD19^+^ B-cells, respectively. 11,971 and 7,882 methylated regions of interest (MRIs) were identified respectively. The genome-wide distribution of these MRIs displayed significant differences between FL and normal B-cells. A reverse trend in the distribution of MRIs between the promoter and the gene body was observed in FL and CD19^+^ B-cells. The MRIs identified in FL cells also correlated well with transcriptomic data and ChIP-on-Chip analyses of genome-wide histone modifications such as tri-methyl-H3K27, and tri-methyl-H3K4, indicating a concerted epigenetic alteration in FL cells.

**Conclusions/Significance:**

This study is the first to provide a large scale and comprehensive analysis of the DNA methylation sequence composition and distribution in the FL epigenome. These integrated approaches have led to the discovery of novel and frequent targets of aberrant epigenetic alterations. The genome-wide bisulfite sequencing approach developed here can be a useful tool for profiling DNA methylation in clinical samples.

## Introduction

Two major processes that contribute to the epigenome of a cell are DNA methylation and histone modifications. Methylation of cytosine residues at CpG dinucleotides is known to regulate gene expression and aberrant promoter hypermethylation has been associated with transcriptional silencing of tumor suppressor genes (TSGs) in various types of tumors including hematological malignancies [1], [2]. Given the important role of DNA methylation in tumor initiation and progression, distinct efforts have been made towards the use of DNA methylation as a biomarker in cancer [3], [4]. In addition, since this epigenetic change potentially is reversible, demethylating agents now are approved for use in the treatment of hematological tumors such as myelodysplastic syndrome [5]. Although lymphomas and leukemias are well characterized by widespread genomic abnormalities such as chromosome translocations, we and others have found that aberrant promoter hypermethylation also is a common event in hematological tumors [6], [7], [8], [9], [10].

Polycomb (PcG) proteins are multiprotein complexes that epigenetically silence gene expression, including many TSGs [11]. PcG proteins exist in at least two separate protein complexes: Polycomb repressive complex 1 & 2 (PRC1 and PRC2). PRC2, consisting of EED, EZH2, YY1 and SUZ12, is thought to be required at the initiating stage of silencing, whereas PRC1, containing HPH, RING1,BMI1, and HPC, is required continuously for the stable maintenance of the initiated PcG repression on specific target loci [12]. EZH2 has histone methyltransferase activity specific for histone H3 lysine 27, and SUZ12 and EED are required for this activity. EZH2 can directly recruit DNA methyltransferases (DNMTs) and lead to *de novo* DNA methylation [13]. EZH2 is known to play an important role in B-cell development and VDJ recombination [14]. Further immunohistochemical studies have revealed that in the germinal center, proliferating centroblasts express certain components of the PRC2 complex, whereas non-proliferating centrocytes and naïve B cells do not [15]. Recent studies have shown that a large group of frequently methylated genes in FL cells were targets of the PRC2 complex in embryonic stem (ES) cells [6], [8], [10]. Although the underlying mechanism is still unclear, dysregulation of polycomb protein expression was reported in lymphomas [15], [16]. It is postulated that germinal center lymphomas such as FL are initiated in the germinal center stage with proliferating cells and elevated polycomb protein expression [7].

In this study, we have integrated the concept of reduced representation bisulfite sequencing (RRBS) with the methylated CGI recovery assay (MIRA) for genome-wide bisulfite sequencing analysis using 454-sequencing technology. We have sequenced the methylome of a FL cell line and normal CD19^+^ B-cells. We also compared the genome-wide methylation patterns with gene expression and histone methylation profiles in FL cells. These integrated analyses identified many novel DNA methylation targets in the FL epigenome and provided a comprehensive analysis of the DNA methylation present within the genome and the distribution of other epigenetic marks.

## Results

### Genome-wide bisulfite sequencing of RL and CD19^+^ B-cell DNA

**Figure 1:**
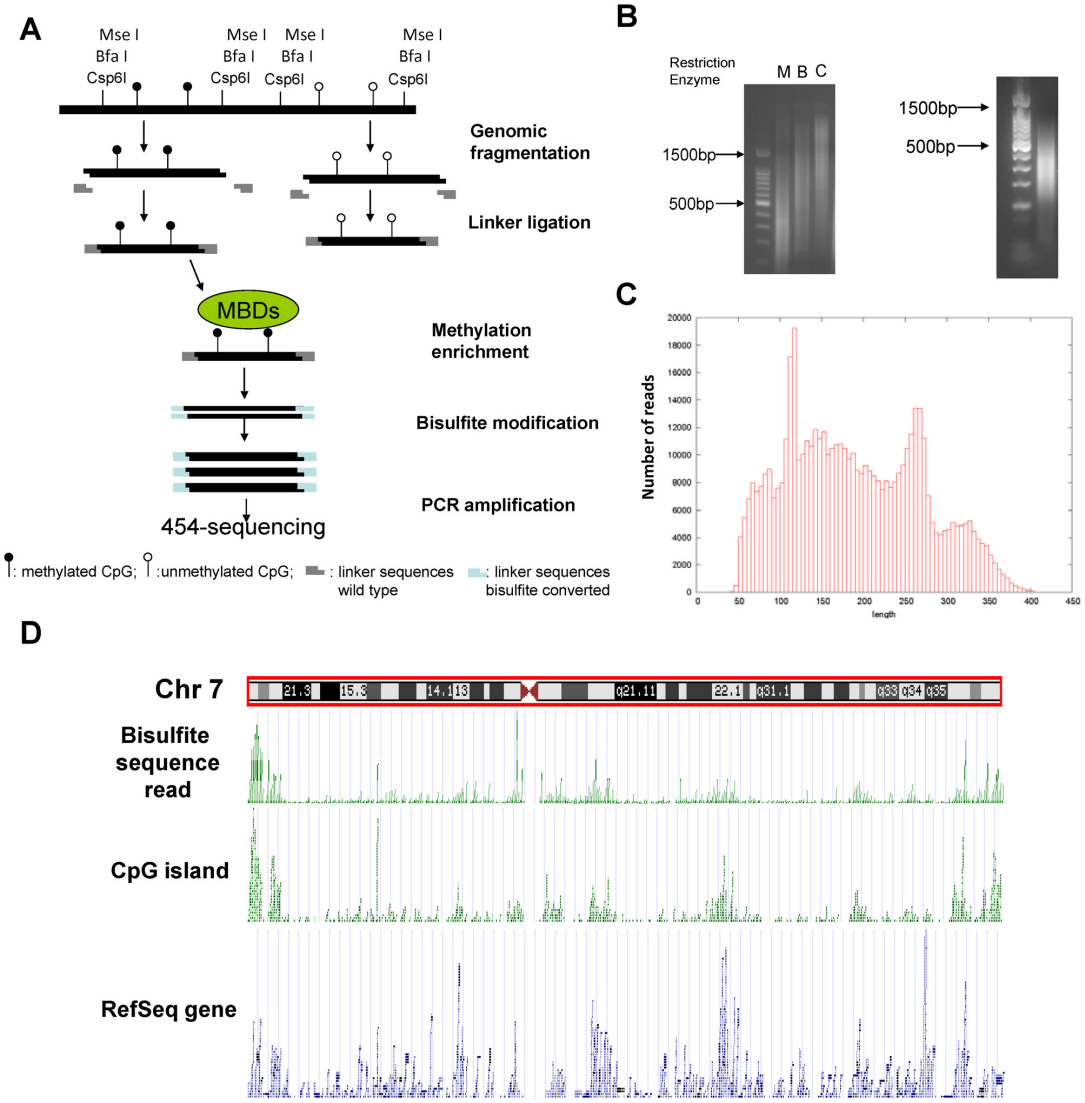
Experimental Design. **A**. Schematic diagram of MIRA-454 sequencing approach. **B**. Left panel: Gel electrophoresis of *Mse* I (M), *Bfa* I (B), and *Csp* 6I (C) digested genomic DNA. Right panel: PCR results of the adaptor-ligated and bisulfite-treated DNA. **C**. Histogram of the read length distribution of bisulfite sequences obtained from 454-sequencing of the methylation enriched genomic library. **D**. Mapping of the bisulfite sequencing reads along chromosome 7. A custom track was generated from alignment results of bisulfite sequences on human genome. The track was uploaded to the UCSC genome browser and compared with other tracks such as RefSeq genes and CpG islands.

Using the bisulfite sequencing strategy illustrated in Figure 1A–B and described in detail in the methods section, we first sequenced the FL cell line, RL. We collected 518,797 bisulfite sequences (approximately 100 Mb) with an average read length of 143 bp after trimming off the adaptor sequences (range 20 bp to 444 bp, see Figure 1C) using the GS FLX sequencer. A novel program called BSmapper was used to align bisulfite sequences to the human genome. After filtering out the sequences mapped to short clusters of length less than 50 bp, 389,038 bisulfite sequences (75% of all reads) were uniquely mapped to the non-repeat-masked human genome (NCBI build 36.1,hg18). Overall 101,631 unique genomic loci were mapped by at least one sequence read and the total number of bases covered in the human genome by all sequencing reads was approximately 18.2 million including 5.3 million cytosines and 726,003 CpGs (Table 1). 78.5% of the 726,003 CpGs sequences contain methylated cytosines. Bisulfite treatment efficiency was determined by calculating the C to T conversion rate for all cytosine bases other than those in CpG dinucleotides (this includes CpA, CpC or CpT dinucleotides and is from this point referred to as CpH). The bisulfite conversion rate was estimated to be 97%, however, it could not be determined if any of the unconverted cytosines were due to *de novo* CpH methylation. Similar to the ChIP-seq method [17], since methyl-binding proteins were used to extract the methylated GC-rich DNA, we observed significant enrichment of sequence reads at CGI regions compared to other genomic regions. Using chromosome 7 as an example (Figure 1D), the alignment results show that the peak of the sequence reads mapped to the genome correlated well with the distribution of CGIs along the chromosome.

**Table 1:** Summary Statistics.

	all		Mapped with > = 5 reads & length >50 bp	
	RL	CD19^+^ B-Cells	RL	CD19^+^ B-Cells
Total number of read	518,797	422,466		
Total number of reads uniquely mapped	389,038	351,015	256,430	82,653
Total number of bases covered in the human genome	18,221,899	49,257,534	3,665,341	3,226,745
Maximal, minimal and average mapped read length after trimming off linker sequences	444/20/156	692/20/218	442/50/158	692/20/228
Total number of cytosines covered in the human genome	5,287,738	12,251,399	1,160,014	895,043
Total number of CpGs covered in the human genome	726,003	1,332,822	227,904	152,846
Total number of mCpGs identified	570,260	1,205,001	198,839	148,205
Total number of genomic loci identified	101,632	207,708	13,082	8,020
Total number of CpG islands sequenced	12,757	4,915	4,479	1,000

We also generated 403,525 bisulfite sequences (approximately 88 Mb) for CD19^+^ B-cells isolated from normal peripheral blood using the GS FLX sequencer, but with the new Titanium chemistry. The average read length was 218 bp after trimming off the adaptor sequences (range 20 to 692 bp). The longer average read length was mainly due to the improved sequencing chemistry. 351,015 bisulfite sequences (83% of all reads) were uniquely mapped (Table 1). The total number of bases covered in the genome was 49.3 million including 12.3 million cytosines and 1.3 million CpGs (Table 1). Nearly 90% of the 1.3 million CpGs contain methylated cytosines. Although the total number of reads uniquely mapped to the genome was similar between RL and CD19^+^ B-cells, the total number of bases or CpGs covered in the genome was significantly higher for CD19^+^ B-cells. The genomic loci identified were nearly doubled for CD19^+^ B-cells.

### Distribution of methylated DNA across the genome in RL and CD19^+^ B-cells

**Figure 2:**
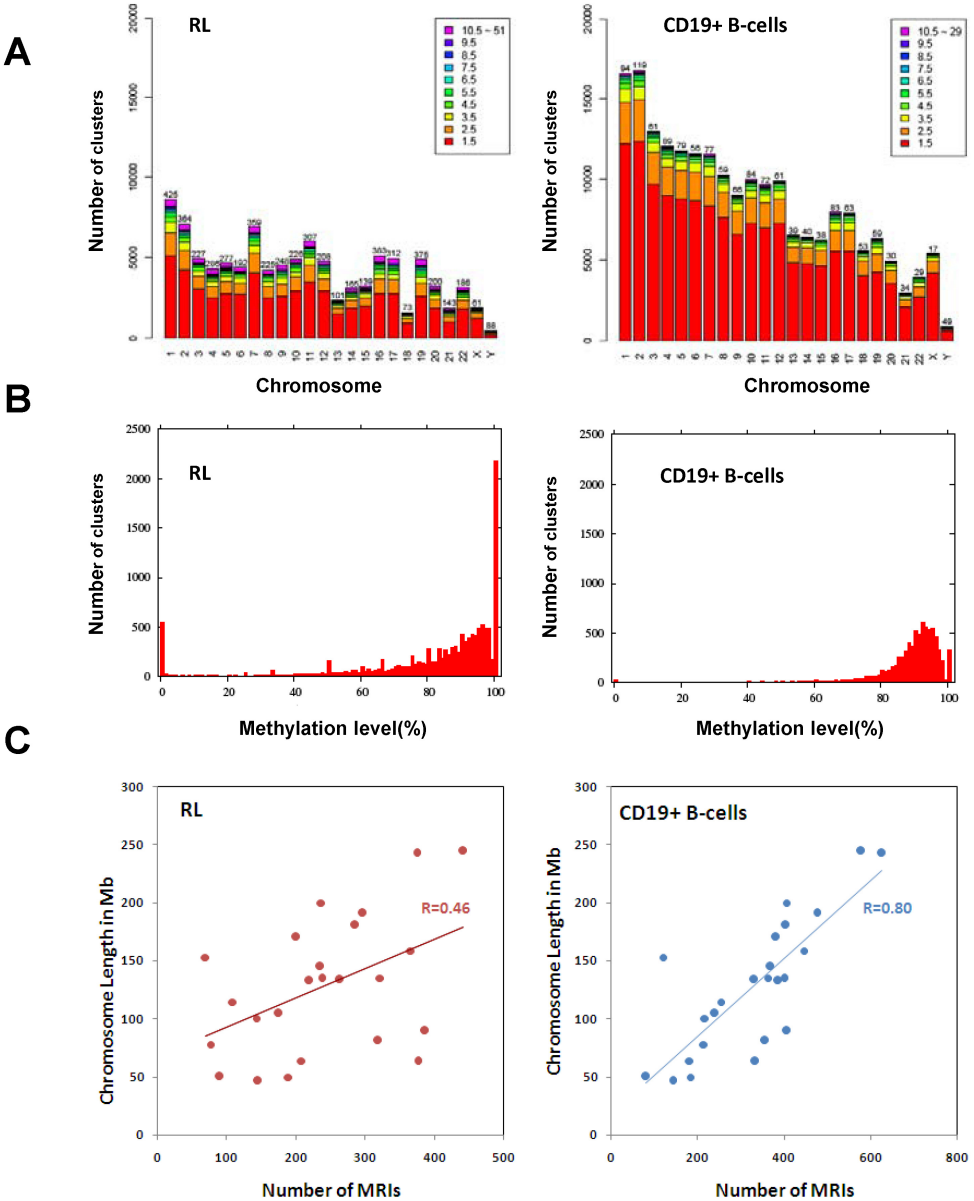
Distribution of bisulfite sequencing reads in the human genome. **A**. Identification of clustered bisulfite sequencing reads on each chromosome. The colors indicate the number of bisulfite sequencing reads mapped to each cluster. Each column indicates the total number of genomic loci mapped with bisulfite sequences on each chromosome. The color scales indicate the number of reads mapped to each locus. The number on the top of each column indicates the number of clusters mapped with 10 or more reads on each chromosome. **B**. The histogram of the distribution of DNA methylation indices across all clusters mapped with at least 5 sequencing reads. **C**. Distribution of MRIs along individual chromosomes. The number of MRIs was plotted against the length of the chromosomes.

As a majority of the genomic loci were mapped with fewer reads (Figure 2A), we established a threshold for identifying clustered regions with multiple overlapping or continuous sequencing reads (≥5) and longer than 50 bp. Using this criteria, we identified 13,082 and 8,020 clusters in RL and CD19^+^ B-cells, respectively (Table 1). The average number of reads per cluster was 19.5 and 10.3, and the maximum number of reads for a given cluster was 1,969 and 2,395 in RL and CD19^+^ B-cells, respectively. The average length of a cluster was 280 bp (range 50 to 2,438 bp) and 402 bp (range 50 to 2,430), respectively. After excluding 318 and 95 clusters in RL and CD19^+^ B-cells that do not contain any CpG sites, we calculated the methylation index for each cluster by averaging the methylation level at each CpG site within each cluster. Figure 2B shows the distribution of methylation indices of CpGs among clusters, with most being largely methylated (>80%). However, 793 and 43 clusters were identified with a DNA methylation index of less than or equal to 20% in RL and CD19^+^ B-cells, respectively.

We defined a methylated region of interest (MRI) if a particular cluster was at least 50 bp long, contained at least 5 mappable reads, and had a methylation index of greater than 20%. Figure S1 shows the distribution of these clusters across the genomes of RL and CD19^+^ B-cells. Independent confirmation studies of several loci using the Sequenom MassArray demonstrated that these parameters were stringent enough for identifying MRIs. Examples of the confirmation studies can be found in Figure S2A–D. We also conducted a permutation test to assess whether the distribution of methylation levels of MRIs was obtained by chance in RL cells. For each permutation, we randomly shuffled the methylation level of all CpGs in our data set, calculated the average methylation level of clusters, and then determined MRIs. Figure S3A shows a histogram of methylation indices after 1000 permutations. Compared to Figure 2B left panel, the number of clusters with 0 or 100% methylation was significantly reduced, suggesting that it was unlikely to obtain the original distribution by chance. A student t-test was performed for each permutation to assess how much a permuted distribution was similar to the original distribution. Figure S3B shows that all *p*-values in 1000 permutations were less than 0.003, indicating that the criteria used to determine MRIs were reasonable.

A total of 11,971 and 7,882 MRIs were identified in RL and CD19^+^ B-cells based on the parameters mentioned above. Similar to the previous report [18], the number of MRIs per chromosome was roughly correlated with the size of the chromosomes in CD19^+^ B-cells (Figure 2C, right panel). Several chromosomes such as chromosomes 16, 17, 19, and 22, had a higher number of MRIs per Mb, and chromosomes X and Y had the lowest number of MRIs per Mb. However, the distribution of MRIs among chromosomes was skewed in RL cells. The Pearson's correlation coefficient between the number of MRIs and the size of the chromosomes dropped by nearly half (Figure 2C, left panel). The number of MRIs on chromosome 13 and 18 dropped more than 2 fold. Rauch et al. (2009) identified more MRIs in normal CD19^+^ B cells than our study [18]. This difference primarily was due to the analytical platforms (microarray vs. sequencing) and the reduced representation by restriction enzymes employed in our study, but not in Rauch's study.

**Figure 3:**
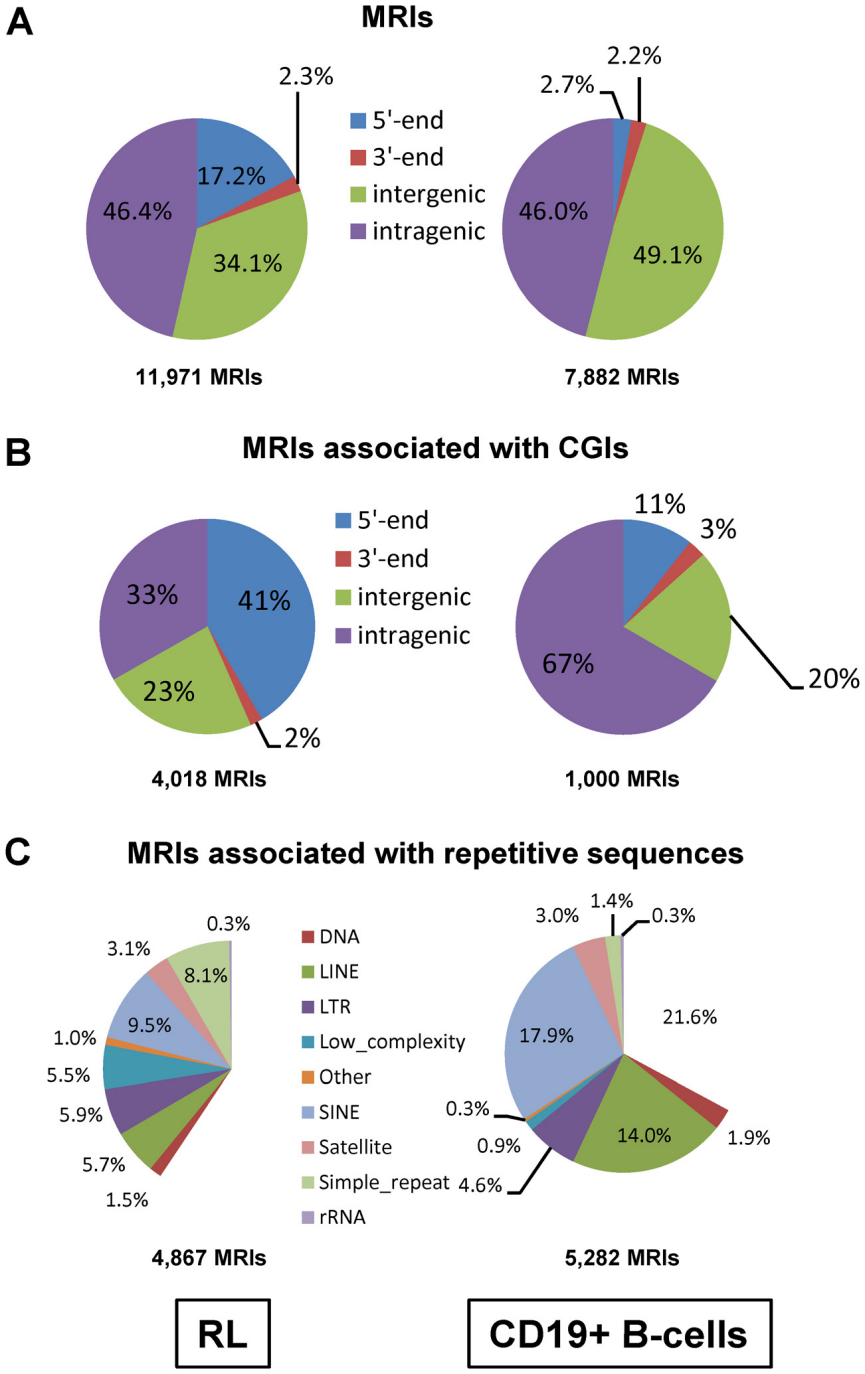
Distribution of MRIs and methylated CpG islands relative to annotated genes. **A**. Pie charts of the distribution of MRIs relative to annotated genes. The MRIs identified in RL and CD19^+^ B-cells were annotated based on their location with respect to genes as described in the text. **B**. Pie charts of the distribution of MRIs associated with CGIs relative to annotated genes. **C**. Pie charts of the distribution of the repetitive sequences in MRIs identified in RL and CD19^+^ B-cells.

In RL cells, 30% of the 11,971 MRIs were located within or overlap with 4,033 CGIs, which accounts for 14.3% of all CGIs annotated in the UCSC genome browser. In general, these MRIs covered four distinct sequence categories based on gene annotation (NCBI Build 36). As shown in Figure 3A left panel, 2,054 (17.2%) of the 11, 971 MRIs were located within the 5′-end of annotated genes (defined as −2,200 bp to +500 bp around the transcription start sites), 5,557 (or 46.2%) were located in the gene body (+500 bp to the transcription start and −500 bp to the transcript end) and 275 (or 2.3%) at the 3′-end of a gene (±500 bp to the transcript end). The list of MRIs located in the 5′-end of the genes can be found in Table S1. 4,086 (or 34.1%) MRIs were located in intergenic regions of the annotated genes. However, for those MRIs associated with CGIs, nearly half of the MRIs were located within the 5′-end of annotated genes (Figure 3B left panel). 4,867 (∼40%) MRIs mapped to regions that contain repeat sequences, and the most abundant repeat sequences were SINEs, simple repeats, LINEs and LTRs (Figure 3C left panel).

In contrast with RL, only 1,000 (12.3%) of the 7,882 MRIs identified in CD19^+^ B-cells were located within or overlap with CGIs; and only 215 (2.7%) of the 7,882 MRIs were located within the 5′-end of annotated genes (Figure 3A right panel). The list of MRIs located in the 5′-end of the genes in CD19^+^ B-cells can be found in Table S2. Most of the MRIs (95%) identified in CD19^+^ B-cells were located in either intra- or intergenic regions. The percentage of MRIs located in the 3′-end of the annotated genes were similar between RL and CD19^+^ B-cells. However, only 11% of the MRIs associated with CGIs were located at the 5′-end of annotated genes in CD19^+^ B-cells and two thirds of them were located within the intragenic regions in CD19^+^ B-cells (Figure 3B, right panel). In addition, 60% of MRIs in CD19^+^ B-cells mapped to the regions that contain repeat sequences (Figure 3C right panel).

Overall, the sequencing results indicated that the distribution of MRIs in RL and normal B-cells was quite different. The MRIs identified in normal B-cells were mostly in intra- or intergenic regions and were associated with repetitive sequences. However, the proportion of MRIs located in the promoter or 5′-end of genes was significantly higher in RL cells, while the number of MRIs located within intra- or intergenic regions decreased as compared to CD19^+^ B-cells. Among 11,971 MRIs identified in RL cells, 1,455 of them overlapped with the 7,882 MRIs identified in CD19^+^ B-cells, accounting for only 12% of the MRIs identified in RL cells. The Pearson's correlation coefficient between the methylation levels of MRIs in RL and CD19^+^ B-cells was also quite low (Figure S4).

### Promoter hypermethylation in RL cells

**Figure 4:**
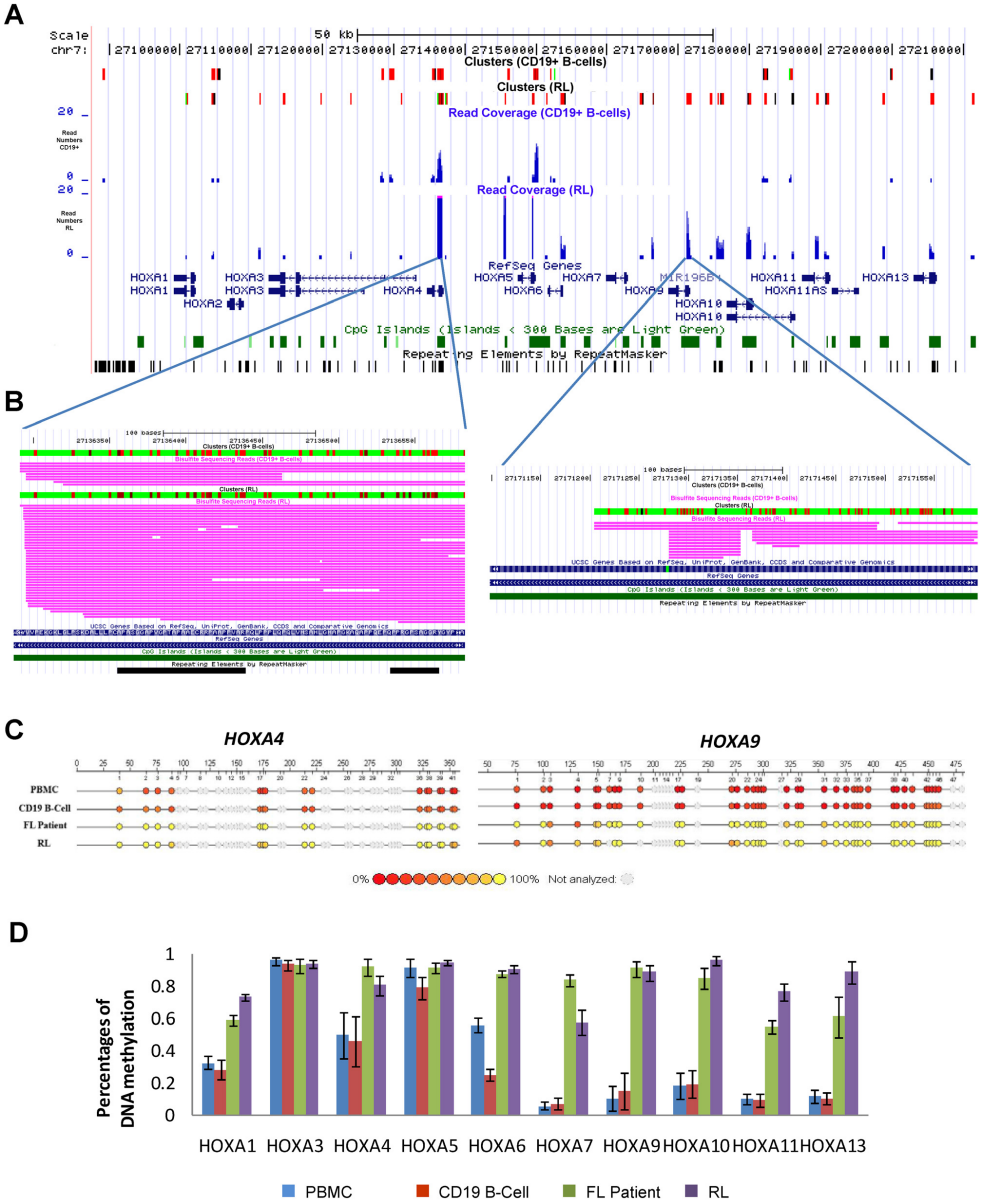
Bisulfite sequencing results of the HOX A gene cluster. **A**. Alignment results of bisulfite sequencing reads in the HOX A gene cluster are shown as custom tracks in UCSC genome browser. The top two tracks show CpG sites that are covered by sequencing reads. The red bars indicate methylated CpGs. The middle two tracks in blue color indicate the coverage of sequencing reads at each CpG site. **B**. A blow-out view of two loci (*HOXA4* and *HOXA9*) mapped with multiple bisulfite sequencing reads were shown in the lower panel. The vertical bar in red color indicates the level of methylation at each CpG site. The deeper red shade indicates the higher methylation level. The horizontal tracks in pink color are the individual bisulfite sequencing reads. **C**. The bisulfite PCR products corresponding to the two regions in panel B were analyzed using the MassArray method. Bisulfite- treated genomic DNA from normal PBMC, CD19^+^ B-cell, a FL patient sample and RL was used as template for PCR. Each circle indicates a CpG site and the methylation level is indicated by color. The methylation level was determined as an average of four independent replicates. **D**. MassArray analysis of 10 HOXA genes is shown as Bar graph. Each bar represents the average methylation level of all CpG sites analyzed by each bisulfite PCR product. Error bar are generate from 4 replicates.

We identified 1,878 and 223 genes that were associated with MRIs in the 5′-end of these genes in RL and CD19^+^ B-cells, respectively (See Table S1 and S2). 59 genes were methylated in both RL and CD19^+^ B-cells; 1,817 and 164 genes were only methylated in either RL or CD19^+^ B-cells, respectively. Since our previous study [10] found that a number of hox genes were methylated in RL cells, we decided to take a look at all of the HOX gene families in RL and CD19^+^ B-cells. Figures 4A-B and S5 show that multiple bisulfite sequencing reads were aligned to over a dozen HOX genes including *HOX A4*, *A5*, *A6*, *A9*, *A10*, *A11*, *A13*, *B1*, *B2*, *B8*, *B9*, *B13*, *C5*, *C9*, *C12*, *D1*, *D3*, *D8* and *D13*. We confirmed *HOXA4* and *HOXA9* promoter CGI methylation in RL cell line, primary FL, and normal CD19^+^ B-cells using the Sequenom MassArray Technology (Figure 4C). The MassArray data demonstrated that 8 out of 10 HOX A genes were methylated at intermediate or lower levels in normal peripheral blood mononuclear cells (PBMCs) and CD19^+^ B cells isolated from PBMCs, but become hypermethylated in FL cells (Figure 4D). Only *HOXA3* and *HOXA5* showed similar levels of methylation in FL and normal CD19^+^ B- cells. Overall, methylation of the HOXA genes in the primary FL tumor and the RL cell line were similar. In addition to HOX gene clusters, we identified methylation in the promoter CGIs of several protocadherin gene families, all of which are located at chromosome 5q21 (See Figure S6). The protocadherin gene family has genomic structure similar to *IGH* and has multiple splicing forms and multiple alternative promoters. Our sequencing data shows that a majority of these promoter CGIs are methylated in both RL cells and CD19^+^ B-cells. Most of these genomic regions span over one hundred thousand to several million base pairs. Methylation seems to play an important role in controlling gene expression of large genomic regions, which is consistent with similar observations from several recent publications [19], [20].

Methylation also was observed in other gene families or pathways that share similar molecular functions. For example, methylation was observed in the promoter of 11 out of 20 *SOX* genes and 7 out of 10 Frizzled protein genes in RL cells. We analyzed the 1,817 genes that were hypermethylated only in RL cells using the DAVID interface (http://david.abcc.ncifcrf.gov/). Using the functional annotation clustering with high stringency, these genes fell within 4 major functional groups including development, glycoproteins, signal transduction, and transcriptional factors (Figure S7). Similar to our previous study using microarray-based analyses, genes for homeobox proteins and the WNT signaling pathway were among the most enriched functional groups [10].

The hypermethylated genes in RL also contained a large number of known genes found to be methylated in primary FLs (See Table S1) by previous studies [6], [7], [8], [10]. We now have detailed DNA methylation maps of these candidate genes in RL. As an example, DNA methylation patterns of a 200 bp fragment located in the first exon of tumor suppressor gene *HIC-1* is shown in Figure S8. There seems to be a gradual decrease in methylation towards the 5′end of the gene. The first 50 bp of this 200 bp fragment contains mostly unmethylated CpGs, while the next 150 bp are predominately methylated. However, this observation may require further validation. We also observed interesting methylation patterns in HLA-A, where a heterozygous polymorphism (rs41558424) can determine the methylation status of adjacent CpG sites (Figure S9). The expression of HLA-A genes also was down regulated in RL cells and can be up-regulated by demethylating treatments (data not shown).

### Correlation of DNA methylation with other epigenetic modifications in RL cells

**Figure 5:**
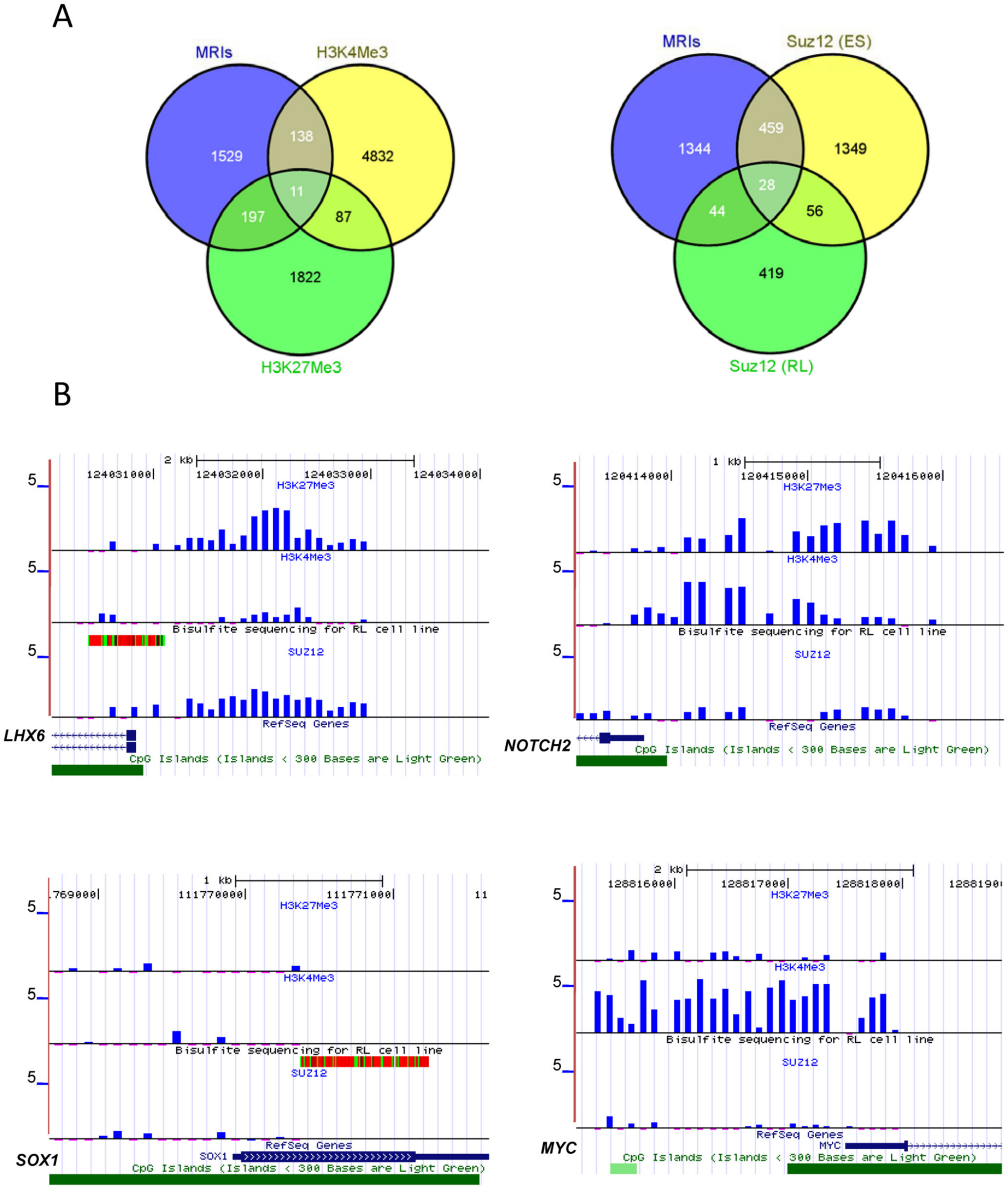
Correlation of DNA methylation with histone methylation and transcriptional repressor binding. **A**. Venn diagrams showing an overlap of MRIs with genes associated with H3K4Me3 and H3K27Me3 marks (*left*) and an overlap of MRIs with SUZ12 target genes in ES cells and RL cells (*right*). **B**. DNA methylation, H3K4Me3, H3K27Me3, and SUZ12 binding profiles in four representative promoters including *LHX6* (*top left*), *NOTCH2* (*top right*), *SOX1* (*bottom left*), and *c-MYC* (*bottom right*). Each blue bar corresponds to the log2 ratio of ChIP/Input DNA for an individual probe. The methylation tracks are the same as described in Figure 4.

Recent studies from our own group and others show that methylated genes identified in FL significantly overlap with PRC2 target genes in ES cells [6], [8], [10]. We therefore queried the list of genes associated with MRIs in the promoter region using ONCOMINE concept map analysis and the results confirmed that the methylated genes identified by 454-sequencing in RL cells significantly overlap the PRC2 target genes in ES cells (Figure S10). To investigate the correlation of DNA methylation with other epigenetic marks we generated genome-wide profiles of histone H3 lysine 4 tri-methylation (H3K4Me3), H3 lysine 27 tri-methylation (H3K27Me3), and SUZ12 binding in RL cells using ChIP-on-Chip assays on the NimbleGen 385K minimal promoter array. This array is designed to interrogate promoter regions, at positions −2,200 bp to +500 bp around the transcription start sites. The ChIP-on-Chip analyses revealed that 5,068 promoters were enriched with H3K4Me3 in RL cells; 2,117 of promoters were enriched with H3K27Me3, and 548 promoters were bound by Suz12 (Table S3, S4, S5). The ChIP-on-Chip results were confirmed independently using ChIP-PCR (Figure S11). The global analyses showed that MRIs do not overlap significantly with either H3K4 or H3K27 tri-methylation in the promoter regions in RL cells (Figure 5A left panel). In contrast to ES cells, we found only 99 bivalent promoters in RL cells and 11 of them co-localize with MRIs. Although 28% of MRIs are PRC2 target genes in ES cells, our ChIP-on-Chip analysis showed that only 13% of MRIs are associated with H3K27Me3 marks and only 5% of the MRIs are bound by Suz12 in RL cells *in vivo* (Figure 5A right panel). Figure 5B shows several examples of the complexity of gene specific epigenetic regulation. SUZ12 binding, histone H3K27 trimethylation and DNA methylation co-localized in the *LHX6* promoter, while *SOX1* seems to be silenced only by promoter DNA methylation. *NOTCH2*, which plays a pivotal role in the development of marginal zone B-cells, had a bivalent promoter. The promoter of the c-*MYC* oncogene was completely occupied by histone H3K4 trimethylation. Gene-ontology analysis showed that H3K27Me3 and SUZ12 regulated a different set of genes in RL cells compared to ES cells. Expression of many lineage specific genes seemed to be controlled by histone modifications. For instance, H3K27Me3 marks were associated with CD34 and CD44 in RL cells, while H3K4Me3 marks were associated with CD19, CD10 (MME), CD27 and CD38.

### Correlation of DNA methylation with gene expression profiles

**Figure 6:**
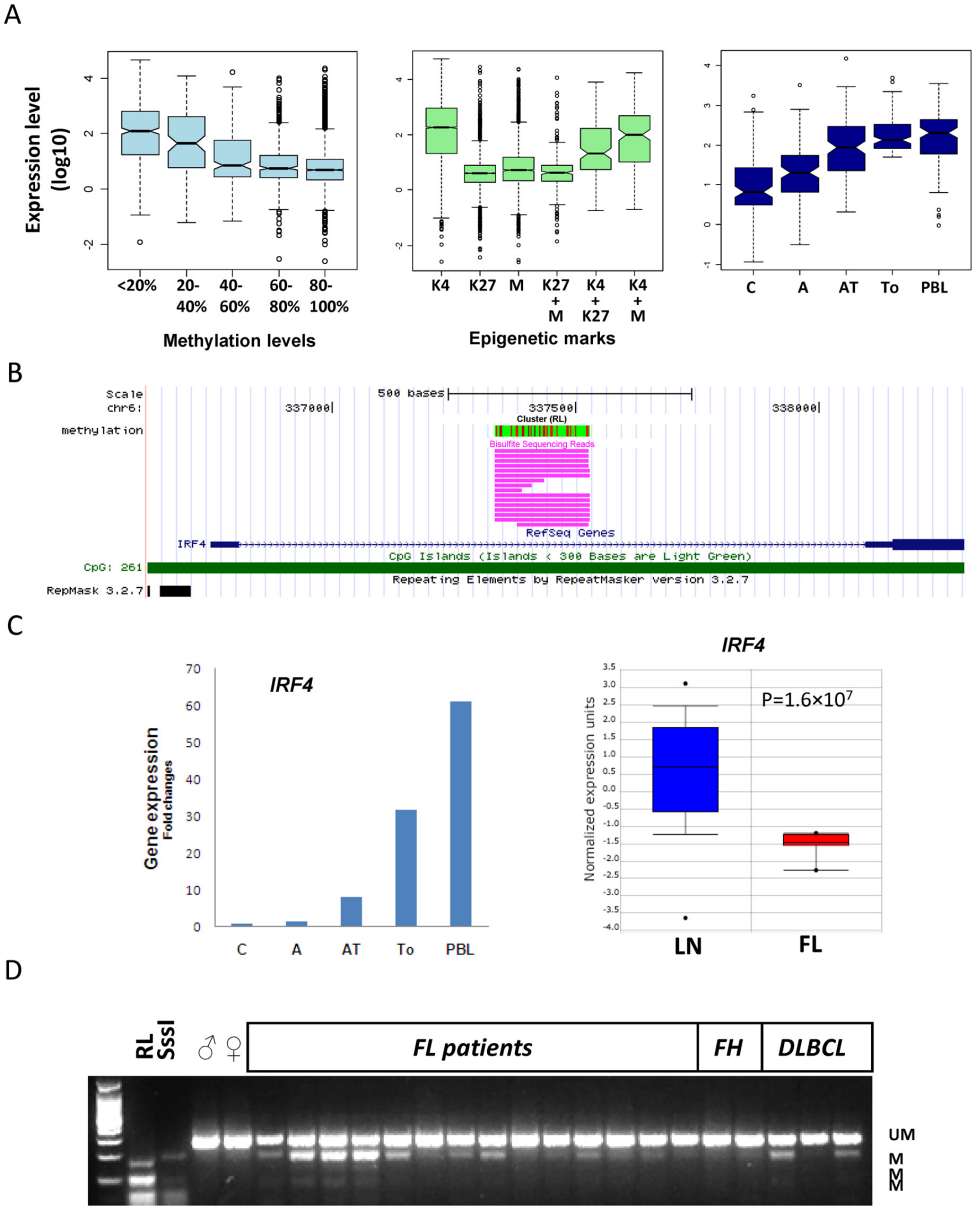
DNA methylation and gene expression. **A**. Box plot showing median expression values of genes associated with MRIs correlate with degree of methylation (*left*), the histone methylation marks (*middle*), and genes associated with MRIs that were down-regulated in RL cells could be re-activated upon treatment of DAC or DAC plus TSA(*right*). The y-axis shows the log10 transformed arbitrary expression value for each individual probe that represents the corresponding transcript. We performed pair wise t-test on the data shown in all three panels and the results can be found in Table S6. Most of the p-values were quite small and demonstrate the differences between groups were significant. **B**. Alignment of bisulfite sequencing reads at 5′ end of *IRF4*. The vertical line in red color indicates the level of methylation at each CpG site, which is the same as described above. The horizontal tracks in pink color are the individual bisulfite sequencing reads. **C**. mRNA expression analysis by microarray shows that *IRF4* is down-regulated in RL cells and can be reactivated by DAC and DAC plus TSA treatment (*left*). The right panel shows *IRF4* expression in normal lymph nodes (blue) and follicular lymphoma (red), as reported in ref [42]. **D**. COBRA analysis of *IRF4* methylation in primary FL, DLBCL, FH and normal control DNA. *Symbols*: C, untreated RL control; A, RL with DAC; AT, RL with DAC plus TSA; To, tonsil CD19^+^ B-cells; PBL, CD19 B-cells isolated from PBMC; LN: lymph node; FH, follicular hyperplasia.

We compared the DNA methylation, histone methylation and previously published gene expression profiles obtained from the RL cell line [10]. mRNA expression analyses of RL cells treated with the demethylating agent, 5′-aza-2′-deoxycytidine (DAC) and the histone deacetylase (HDAC) inhibitors, Trichostatin A (TSA), and normal CD19^+^ B cells from tonsil and PBMCs were conducted using Illumina whole genome bead arrays [10]. 1,831 of the 25,400 RefSeq genes have MRIs mapped in the promoter or 5′ regulatory regions. In general, the expression of these genes was correlated with level of DNA methylation of MRIs (Figure 6A left panel). The median value of absolute gene expression was much higher for the genes with lower DNA methylation levels. 4,510 and 1,935 genes had H3K4Me3 and H3K27Me3 marks enriched in their promoter, respectively and the gene expression levels also correlated well with histone modifications overall. The genes associated with promoter H3K4Me3 methylation displayed higher median expression levels even if DNA methylation or H3K27Me3 marks co-existed. On the contrary, genes associated with promoter DNA methylation or H3K27Me3 alone and in combination had lower median expression levels (Figure 6A middle panel). Expression array analyses also revealed that a group of genes that were down regulated in RL cells could be reactivated by epigenetic drug treatments (Figure 6A right panel). Interestingly *IRF4*, a tumor suppressor gene in lymphoma and a repressor of *BCL6* transcription was methylated in RL cells (Figure 6B) and down regulated in RL cells (Figure 6C left panel) and primary FL cells (Figure 6C right panel). *IRF4* could be reactivated by DAC and co-treatment with TSA, suggesting to a synergistic reactivation effect (Figure 6C left panel). Further analysis using COBRA showed that methylation in the same locus was identified in primary FL and diffuse large B cell lymphoma (DLBCL) patients' DNA samples, but not in follicular hyperplasia (FH) and normal control male and female (PBMCs) DNA (Figure 6D).

## Discussion

The power of next-generation DNA sequencing technology such as, Roche/454 GS FLX, Illumina Genome Analyzer and ABI SOLiD, is transforming the landscape of epigenomic research. New epigenomic applications such as ChIP-Seq and genome-wide bisulfite sequencing (BS-Seq) have emerged. We previously performed ultra-deep sequencing analysis of bisulfite-modified lymphoma DNA using 454-sequencing [21]. Since then, bisulfite treatment and high-throughput massively parallel sequencing has been used successfully to map a complete methylome of *Arabidopsis* [22], [23] and most recently the methylome of two human cell lines [24]. Although nearly complete sequencing of the methylome of human cells was an outstanding achievement, the whole-genome shotgun approach is too costly to be applied to analyze multiple disease related samples. Meissner et al. described a large-scale random approach termed reduced representation bisulfite sequencing (RRBS) for analyzing and comparing genomic methylation patterns using Illumia/Solexa sequencing [25]. This approach used a restriction enzyme and a size selection step to generate and sequence a defined fraction of a large genome. Zeschnigk et al. applied a similar approach using 454-sequencing with a combination of 4 different restriction enzymes [26]. Several other groups have also developed methods for targeted genome-level bisulfite sequencing [27], [28], [29]. However, these approaches are quite complicated and can achieve substantially lower genomic coverage, making these technologies more suitable for validating findings than for initial discovery [30].

In this study, we described a simple method for the selective sequencing of methylated DNA using next generation sequencing instruments. Using MIRA coupled with 454 sequencing, we demonstrated a highly efficient capture of methylated DNA followed by in-depth sequencing of a large number of CpG islands. The longer bisulfite sequences obtained by 454-sequencing made sequence alignment more accurate and reliable. Above 75% of the bisulfite sequence reads were uniquely mapped and produced meaningful methylation data. This mapping rate of bisulfite sequences was significantly higher than those obtained by Illumina short read sequencing platform, which usually varies significantly between samples [30]. Although the approach we used was biased toward methylated GC-rich DNA sequences, interesting DNA methylation patterns were discovered. For instance, a heterozygous polymorphism (rs41558424), located within a CpG island in the HLA-A gene cluster, was correlated with the methylation status of two neighboring CpG sites (Figure S9). This interesting methylation pattern obtained in the HLA-A region demonstrated that read length matters. Although the bisulfite sequencing results obtained by short read sequencing can adequately quantify the methylation levels, they will not provide single molecule methylation patterns. The methylation patterns observed in Supporting Figure S8 and S9 might not be revealed clearly if short read sequencing were performed.

For the first time, we generated genome-wide, single base-pair resolution DNA methylation maps in FL and CD19^+^ B-cells. These epigenetic “maps” should facilitate a greater understanding of the role of epigenetic modifications in FL. Our results demonstrate that DNA methylation changes in FL are profound and support the notation that aberrant methylation pattern in tumors consists of a global hypomethylation, in conjunction with localized hypermethylation in CpG islands [2]. Recent studies also show that gene body methylation is associated with active gene expression [27]. Interestingly, for those methylated CGIs, we observed a reverse trend between promoter and gene-body methylation in RL and CD19^+^ B-cells. Significantly more (2054 vs. 215) MRIs were identified in the promoter and 5′-end of genes in the FL cell line compared to CD19^+^ B-cells, suggesting a strong shift towards gains in hypermethylation of promoter CGIs in FL cells. The sequencing results also showed that most of the MRIs identified in CD19^+^ B-cells were located in the intra- and intergenic regions and were associated with repetitive sequences. Particularly, over two thirds of methylated CGIs in CD19^+^ B-cells were located in the gene body. These numbers decreased in the FL cells, suggesting hypomethylation in the intra- and intergenic regions. Although these findings are not completely novel, this is the first time that these phenomena are examined in such a large scale and at a single molecule level in FL cells.

Previously, we have found a large number of methylated genes in RL cells as well as primary FL patient samples using microarray approaches [9], [10], [31], which suggest that RL is a good *in vitro* model for studying DNA methylation in FL. Using the massively parallel bisulfite sequencing approach, we confirmed a large number of methylated genes in RL cells, and identified distinguishing methylation patterns in the promoters of these genes. Gene specific methylation patterns for many tumor suppressor genes such as *HIC-1*, *CDKN1C (p57)*, and *IRF4* were established. Interestingly, *IRF4* is a tumor suppressor gene in lymphoma and functions as a suppressor of *BCL6* transcription [32]. Our data suggest that hypermethylation of the *IRF4* gene might be one of the epigenetic mechanisms of *IRF4* down-regulation, which could then lead to *BCL6* over-expression. We also established the DNA methylation patterns of HOX gene clusters. The MassArray data further confirmed the increased methylation in RL cells and primary FL patient samples compared to normal B- cells, suggesting that hypermethylation of HOX genes was tumor specific. The functional consequences of hypermethylation in these HOX genes are largely unknown and further investigation into the extent of aberrant methylation in HOX gene clusters in primary FLs will be very interesting. The large data set generated in this study can become a great resource for future studies of these candidate genes. Our data can serve as a guide for the identification of specific methylated regions of interest and design of gene-specific methylation assays.

Previous studies showed that a large number of PRC2 target genes were hypermethylated in FL cells [6], [8], [10]. However, combined with the ChIP-on-Chip analysis, we found that only a fraction of those target genes were bound by SUZ12, one of the major components of the PRC2 complex. We chose SUZ12 instead of EZH2 for the ChIP-on-Chip analysis mainly due to the availability of ChIP-grade antibodies and previously published ChIP-on-Chip data set [33]. Consistent with the findings of Squazzo et al. [34], the PRC2 complex binds a different set of genes in somatic cells compared to ES cells (Figure 5A right panel). In addition, H3K27Me3 marks and SUZ12 binding in RL cells did not overlap significantly with MRIs (Figure 5A), indicating that H3K27Me3 may suppress the expression of a number of genes without involving DNA methylation. This is consistent with a previous study in solid tumor cell lines [35] and could be caused by the switching of polycomb repressive marks between normal and neoplastic cells [36].

In summary, we have developed a simple method for genome-wide bisulfite sequencing analysis of methylation-enriched DNA. The experimental protocols and the associated bioinformatic software can be used for analyzing clinical samples. Using this novel method, we conducted genome-wide mapping of combined DNA methylation, H3K27Me3 and H3K4Me3 marks in lymphoma cells to get an even more detailed insight for epigenetic regulation in FL tumor cells. With dramatically increased interest and coordinated community based efforts to decode the human epigenome, a comprehensive picture of functional groups that define gene expression patterns in normal and malignant cells is emerging. The hierarchy of epigenetic regulation as described here might facilitate an overall understanding of coordinated regulation of gene expression in cancer and facilitated identification biomarkers for earlier diagnosis, and molecular targets for cancer therapy.

## Methods

### Tissue samples and cell cultures

Anonymous tumor and normal tissues were obtained from the tissue bank established at Ellis Fischel Cancer Center, Columbia, MO. 14 FL, 3 DLBCL, 2 FH and 2 whole blood samples were used in this study. Histopathological classifications were according to the guidelines of the World Health Organization (WHO). The study was reviewed and approved by the Health Sciences Institutional Review Board of the University of Missouri. PBMCs were isolated using ficoll gradient centrifugation. CD19^+^ B-cells were isolated with magnetic Dynabeads™ (Invitrogen, Carlsbad, CA). The FL cell line, RL, was purchased from ATCC (Catalog No. CRL-2261) and maintained in RPMI 1640 supplemented with 10% FBS [31]. Gene reactivation experiments with DAC, and TSA were described previously [10]. DNA and RNA were isolated using the QIAmp DNA Blood Mini kit and RNeasy kits respectively (Qiagen, Valencia, CA).

### Preparation of linker ligated genomic DNA library

Genomic DNA (2 µg each) was digested to completion by overnight incubation with *Mse* I, *Bfa* I, and *Csp* 6I (New England Biolabs, Ipswich, MA), respectively (See Figure 1B left panel). The digested fragments share a common feature; a TA overhang is present at both ends of each fragment. Therefore these fragments can be ligated to the same adapter. DNA was purified and *Mse* I, *Bfa* I, and *Csp* 6I fragments mixed in equal proportions were pooled together, and then ligated to adapter pre-annealed from oligonucleotides 5′-AGTTATTCTGGACTGTCGAA GCTGAATGCCATGG-3′ and 5′- pTACCATGGCATTCAGCTTCGACAGTCCAGAAT-3′ in 50 µl volume containing 1600 U T4 DNA ligase (New England Biolabs, Ipswich, MA) for 16 hr at 14°C. Excess adapters were removed using an ultrafiltration column (Millipore, Billerica, MA).

### MIRA

For enrichment of methylated CGIs, MIRA was performed as described previously [37], [38]. Four MIRA binding reactions were performed with 500 ng of each adaptor-ligated genomic DNA samples and incubated with MDB2b and MBD3L1 complexes overnight at 4°C on a rocking platform. After washing the pelletted Sepharose beads three times with binding buffer containing 700 mM NaCl, the methylated DNA–enriched genomic DNA fraction was eluted by addition of guanidinium hydrochloride–containing buffer and purified using QIAquick PCR purification kits according to the instructions of the manufacturer (QIAGEN, Valencia, CA).

### Bisulfite-PCR amplification

The captured DNA (40 ng) from MIRA experiments were treated with bisulfite using the EZ Gold DNA methylation kit (Zymo Research, Orange, CA) and eluted in 10 µl of water. The bisulfite treated DNA was then amplified using forward primer (5′-TTGGATTGTTGAAGTTG AATG-3′), and reverse primer (5′-AAACTATCAAAACTAAATACCATAATA-3′). These primers were designed to match the bisulfite converted linker primer sequences used in the ligation step [39]. The PCR reaction was performed in 50 µl volume, each containing 5 µl bisulfite-treated DNA, 25 pmol of each PCR primer and 2.5 U PfuTurboCx Hotstart DNA polymerase (Stratagene, La Jolla, CA). Touchdown PCR was performed at annealing temperatures from 55 to 52°C (two cycles at each temperature) followed by 10 cycles at an annealing temperature of 51°C. Denaturation (94°C), annealing and extension (72°C) times were 10 s, 30 s and 3 min, respectively. The PCR products were purified with QIAquick columns and amplified again for 10 cycles using an annealing temperature of 55°C. The size of linker-PCR products ranges from 200 bp to 800 bp with an average of 300 bp (Figure 1B right panel). The PCR product was purified using AMPure beads (Agencourt, Beverly, MA) to remove sub-quality fragments (<200 bp) and then used for 454-sequencing.

### Sequencing using 454-GS-FLX

The purified PCR products were end-repaired and ligated to the sequencing adaptors using 454 library construction kits and sequenced according to the manufacturer protocols (454 Life Sciences, Branford, CT).

### Methylation validation

Validation was performed for selected genes using MassARRAY® EpiTYPER (Sequenom, San Diego, CA) on bisulfite-converted DNA according to the manufacturer's standard protocols. The COBRA analysis was conducted as described previously [31].

### Expression microarrays

Illumina Sentrix 6 microarrays (Illumina, San Diego, CA) were used for gene expression analysis. Labeling and hybridization were carried out according the manufacturer's standard protocols. Gene expression data was analyzed using BeadStudio (Illumina, San Diego, CA). Functional annotation and analysis were conducted using Database for Annotation, Visualization and Integrated Discovery (DAVID) v6.7 [40].

### ChIP and ChIP-on-Chip

ChIP experiments were carried out using a Magna ChIP kit (Millipore, Billerica, MA) following the manufacturer's suggested protocols. For each ChIP experiment, 5×10^6^ RL cells were crosslinked with 10% formaldehyde at 37°C for 10 minutes. The following antibodies were used: anti-trimethyl-H3K27 (Upstate, no. 07-449), anti-trimethyl-H3K4 (Upstate, no. 04-745), and anti-SUZ12 (Abcam, ab-12073). For hybridization to the microarrays, 10 ng each of Input and ChIP DNA samples was amplified using GenomePlex WGA kit (Sigma-Aldrich, St. Louis, MO). The Input and ChIP samples were labeled with Cy3 and Cy5 fluorescent dye and co-hybridized on to the NimbleGen promoter microarray (Roche-NimbleGen, Madison, WI) according to manufacturer protocols. The array data were extracted according to standard operating procedures by NimbleGen Systems Inc. The peaks were identified with a cut-off of false discovery rate (FDR) of <0.05 using NimbleGen SignalMap software. The NimbleGen GFF files were then converted into Wiggle format and uploaded to the UCSC Genome Browser for visualization.

### Sequencing data analysis

The sequence reads were mapped to the human genome by an in-house mapping program, called BSmapper (http://sourceforge.net/projects/bsmapper), using suffix array and bit-parallelism, leveraging our previous work on genome assembly and genome alignment algorithms [41]. We divided a mapping process into two phases to model possible bisulfite conversion, i.e., C to T and G to A. For each phase, we converted Ts (As) to Cs (Gs) in reads and reference sequences. A suffix array was built to index the converted reference sequence. We searched the suffix array for a seed (word) of a converted read. We filtered the seed if it contained unallowed cases, i.e., matching T (A) to C (G) from the reference sequence to the seed. Given a set of seeds, we merged seeds if they were near in both reference genome and read. Finally, we aligned flanking regions of seeds and chose the best alignment. BSmapper can be compiled and run in various UNIX environments including MAC OS X and Cygwin for MS-Windows. Pairwise alignments between sequence reads and the reference genome were used for generating clusters with multiple sequence alignments using BioPerl. We built custom tracks of the alignment results that can be uploaded to the UCSC genome browser. The MRIs were compared with UCSC genes, RefSeq genes, CpG islands, and RepeatMasked regions. The corresponding data can be accessed at http://people.cgb.indiana.edu/jeochoi/methylation. By clicking on the names of the read in UCSC genome browser, one can download the sequence from the “External Link” that is linked to our bisulfite-sequence database. All the sequences aligned to the same cluster were visualized using a multiple sequence alignment program. The methylated and unmethylated CpGs were highlighted using different colors. Bisulfite conversion rate was calculated as the number of genomic cytosines outside a CpG context that were unconverted, divided by the total number of cytosines outside a CpG context.

### Determination of the methylation index for each cluster

We defined a cluster as a region that was mapped with more than 5 overlapping or continuous reads. To calculate the methylation index of a cluster, the methylation status of each CpG site in each sequence read was first determined based on a C to T conversion at each CpG site on the forward strand and a G to A conversion on the reverse strand. The percentage of methylation at each CpG site within each cluster was calculated based on the number of sequences containing methylated CpG sites versus the total number of sequences analyzed. Finally, the methylation index for each cluster was calculated by averaging the methylation levels of all CpGs within each cluster. The methylation statuses of nearby CpGs are usually correlated; particularly in this case, where the clusters were identified through specific capture of methylated DNA. Therefore, it is reasonable to group the nearby CpGs and calculate the average methylation levels. The methylation indices allowed us to identify those regions that are significantly methylated and eliminate the false positives.

### Data access

The raw sequencing data has been submitted to NCBI sequence read archive website (http://trace.ncbi.nlm.nih.gov/Traces/sra_sub/) and the accession number is SRA010812.4. The NimbleGen microarray data from this study have been submitted to the NCBI gene Expression Omnibus (http://www.ncbi.nlm.nih.gov/geo) under accession no. GSE20019. The bisulfite sequence tracks in UCSC genome browser format are available on our website (http://people.cgb.indiana.edu/jeochoi/methylation.) The source code package with documentation is available online at http://sourceforge.net/projects/bsmapper.

## Supporting Information

Figure S1 Distribution of MRIs in the genomes of RL and CD19+ B-cells. Figure was generated using the Genome Graphs, a built-in function of the UCSC genome browser. Each dot indicates a MRI as defined in the main text. Blue: RL; Red: CD19+ B-cells. The light green area indicates genomic regions where no MRI was identified in either sample. The Y-axis indicates the methylation indices (a value of 0 to 1) of each MRI.
(3.20 MB TIF)

Figure S2 Sequenom MassArray analyses validating the 454 sequencing results of four representative genes, A-D. For each gene, the top panel shows the 454 bisulfite sequencing results. The description of the track is same as described in the paper. The bottom panel shows the results obtained from the MassArray analysis. Bisulfite- treated genomic DNA from normal PBMC, CD19 B-cell, a FL patient sample and RL cells was used as the template for PCR. Each circle indicates a CpG site and the methylation level is indicated by color. Four technical replicates were analyzed for the each sample.
(5.84 MB TIF)

Figure S3 Results from a permutation test which examines whether the distribution of methylation levels of clusters was obtained by chance. A. Average methylation levels of clusters in 1000 permutation. The Y-axis represents the average number of MRIs for a bin of methylation levels and the error bars represent standard deviation. B. Histogram of p-values in 1000 permutation. The p-values were calculated by a student t-test between the original and permutated distributions. C. Histogram of MRIs in 1000 permutation. After discarding clusters of equal or less than 20% methylation, MRIs were determined in each permutation. The minimum, mean, and maximum numbers of MRIs are 12,369, 12,684, and 12,715. The distribution was far from 11,971 and demonstrated that the distribution was not obtained by chance.
(2.05 MB TIF)

Figure S4 Comparison of the methylation levels of overlapping CpG sites between RL and CD19+ B-cells. A Pearson correlation coefficient of 0.33 was observed between the two samples, suggesting significantly differential methylation.
(0.66 MB TIF)

Figure S5 DNA methylation and histone modification profiles of 4 Hox gene clusters in RL cells. Each blue bar corresponds to the log2 ratio of ChIP/Input DNA for an individual probes. The methylation tracks are the same as described in the paper.
(1.65 MB TIF)

Figure S6 Bisulfite sequencing results of protocadherin gene clusters. A. Alignment results of bisulfite sequencing reads in the PCDHGA-B gene clusters. B. Alignment results of bisulfite sequencing reads in the PCDHA gene clusters. Custom tracks were uploaded into the UCSC genome browser. The vertical line in red color indicates the level of methylation at each CpG site. The deeper red shade indicates the higher methylation level. The blue color peaks show the sequencing coverage for each cluster.
(3.26 MB TIF)

Figure S7 Functional analysis of genes associated with MRIs in the 5′-end. Enrichment scores from analysis of 1,817 annotated genes hypermethylated in RL using DAVID (http://david.abcc.ncifcrf.gov/). The x-axis shows the percentage of methylated genes that fall into functional groups. y-axis shows the functional group. The p-value represents the likelihood that a group of genes are NOT enriched by chance.
(0.14 MB TIF)

Figure S8 The HIC-1 gene, an example of bisulfite sequencing alignment results. The custom track on the top panel shows the methylation levels. Red color indicates the methylated CpGs. The lower panel shows the externally linked multiple alignment results. The methylated and unmethylated CpGs are also highlighted using different colors.
(0.53 MB PDF)

Figure S9 The HLA-A gene, another example of bisulfite sequencing alignment results. The custom track on the top panel shows the methylation levels. Red color indicates the methylated CpGs. The lower panel shows multiple alignment results. The methylated and unmethylated CpGs are also highlighted using different colors. A heterozygous polymorphism (rs41558424) seems to correlate with the two unmethylated CpG sites that are 30 to 50 bp downstream of the SNP.
(4.15 MB TIF)

Figure S10 Oncomine concepts map of methylated genes in RL compared to the known polycomb target genes in ES cells. Node represent molecular concepts (biologically related gene sets. Node size is proportional to the number of genes in the concept. Each edge represents a significant enrichment (p<0.05).
(1.26 MB TIF)

Figure S11 Mapping of Suz12, H3K4Me3 and H3K27Me3 to their target promoters. The NimbleGen promoter oligonucleotide array was hybridized with amplicons prepared from ChIP experiments using antibodies against SUZ12, H3K4Me3 and H3K27Me3, and the input control. Shown are three promoter regions: two that were repressed by SUZ12 (PAX7 and LHX5) and one that is not repressed by SUZ12 and actively transcribed (c-Jun). The fold enrichment was calculated by dividing the SUZ12 or H3K4Me3 and H3K27Me3 hybridization intensity signal by the input control signal for each oligonucleotide probe. Each green bar corresponds to the log2 ratio of ChIP/Input DNA for an individual probes. The inserts within each graph show independent ChIP confirmation using PCR analysis. The primers used in the PCR analysis were designed to span the region (blue horizontal bar) showing the highest peak of enrichment for each promoter.
(0.85 MB TIF)

Table S1 List of MRIs associated with gene promoters in RL cells.
(1.08 MB XLS)

Table S2 List of MRIs associated with gene promoters in CD19+ B-cells.
(0.06 MB XLS)

Table S3 List of genes marked by H3K4Me3 in the promoter regions in RL cells.
(1.33 MB XLS)

Table S4 List of genes marked by H3K27Me3 in the promoter regions in RL cells
(0.57 MB XLS)

Table S5 List of SUZ12 target genes in RL cells
(0.17 MB XLS)

Table S6 p value of pair wise t-test results. The gene expression data in each column of the three panels in Figure 6A was used to conduct the test.
(0.03 MB XLS)
