# Correction: Genome-Wide DNA Methylation Maps in Follicular Lymphoma Cells Determined by Methylation-Enriched Bisulfite Sequencing

**DOI:** 10.1371/annotation/ef58e5ee-1aaf-47b8-a0c5-1f1bf6886a8c

**Published:** 2011-02-25

**Authors:** Jeong-Hyeon Choi, Yajun Li, Juyuan Guo, Lirong Pei, Tibor A. Rauch, Robin S. Kramer, Simone L. Macmil, Graham B. Wiley, Lynda B. Bennett, Jennifer L. Schnabel, Kristen H. Taylor, Sun Kim, Dong Xu, Arun Sreekumar, Gerd P. Pfeifer, Bruce A. Roe, Charles W. Caldwell, Kapil N. Bhalla, Huidong Shi

In the Funding section, one of the grant numbers from the National Cancer Institute was listed incorrectly. The grant number "CA 132706" should be "CA 134304."

