# Supplementary figures and images for: Genome-Wide DNA Methylation Maps in Follicular Lymphoma Cells Determined by Methylation-Enriched Bisulfite Sequencing
Source: PLoS One. 2010 Sep 29;5(9):e13020. doi: 10.1371/journal.pone.0013020 (PMC2947499; doi:10.1371/journal.pone.0013020)

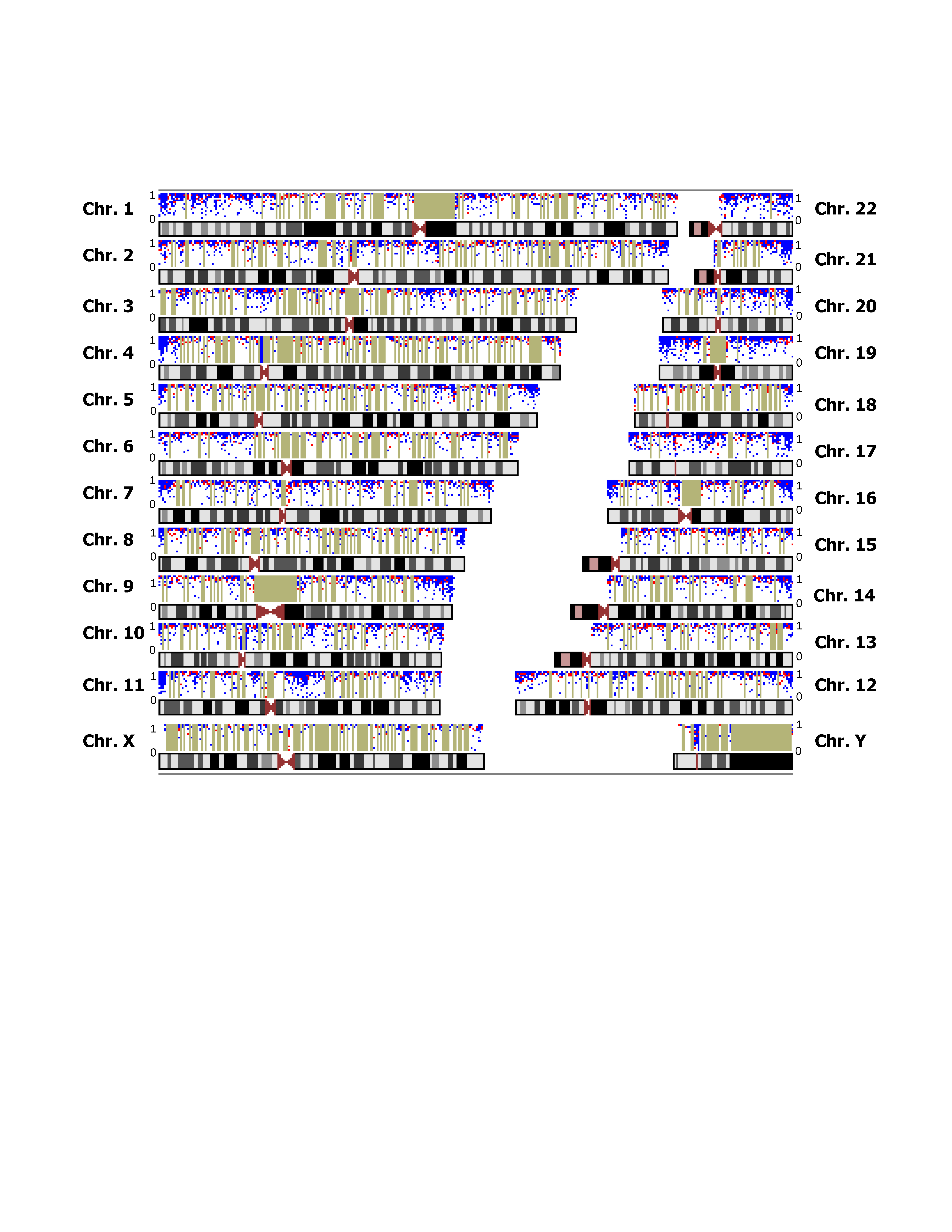

Supplement: Figure S1 — Distribution of MRIs in the genomes of RL and CD19+ B-cells. Figure was generated using the Genome Graphs, a built-in function of the UCSC genome browser. Each dot indicates a MRI as defined in the main text. Blue: RL; Red: CD19+ B-cells. The light green area indicates genomic regions where no MRI was identified in either sample. The Y-axis indicates the methylation indices (a value of 0 to 1) of each MRI. (3.20 MB TIF) [file pone.0013020.s001.tif]

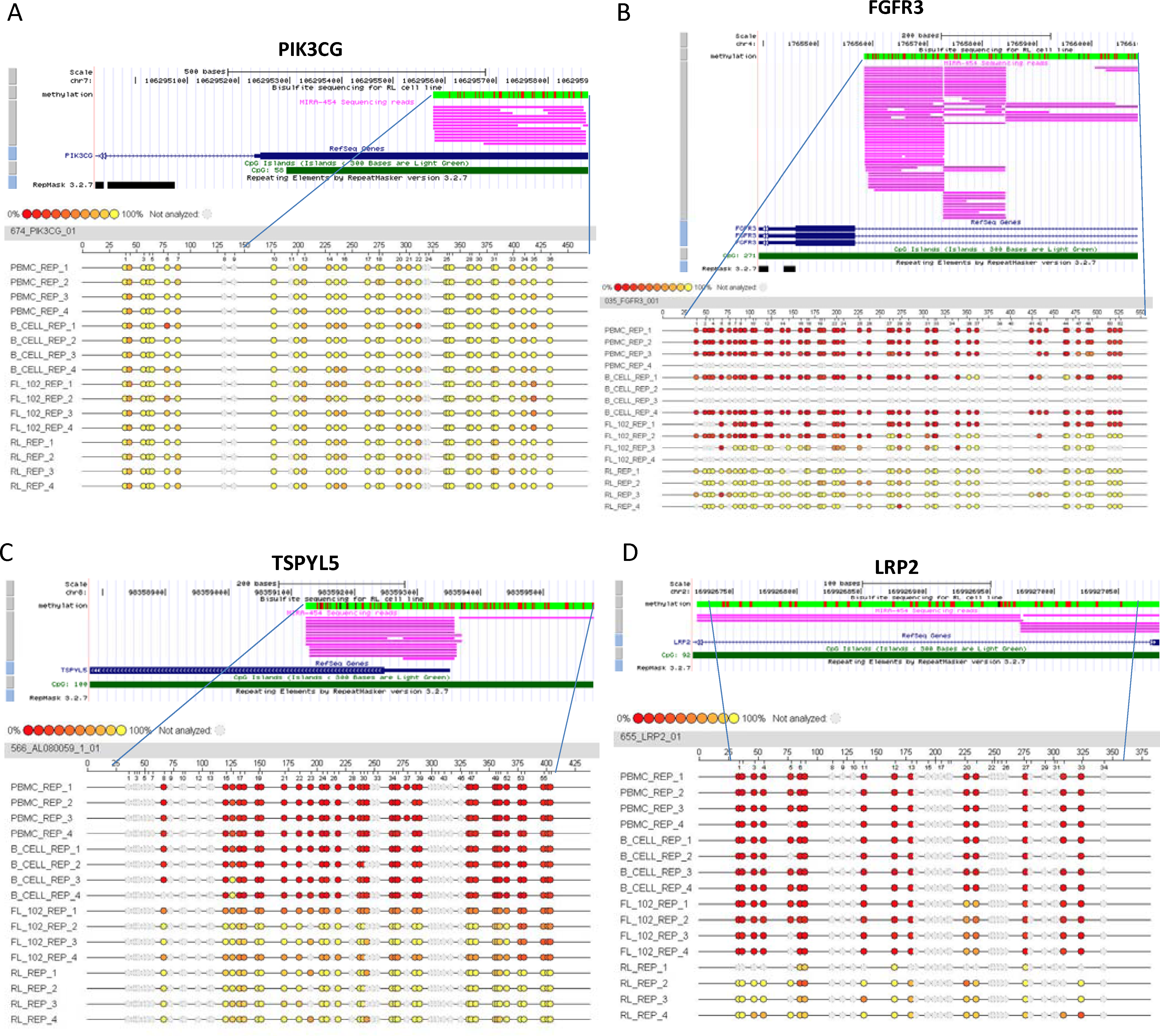

Supplement: Figure S2 — Sequenom MassArray analyses validating the 454 sequencing results of four representative genes, A-D. For each gene, the top panel shows the 454 bisulfite sequencing results. The description of the track is same as described in the paper. The bottom panel shows the results obtained from the MassArray analysis. Bisulfite- treated genomic DNA from normal PBMC, CD19 B-cell, a FL patient sample and RL cells was used as the template for PCR. Each circle indicates a CpG site and the methylation level is indicated by color. Four technical replicates were analyzed for the each sample. (5.84 MB TIF) [file pone.0013020.s002.tif]

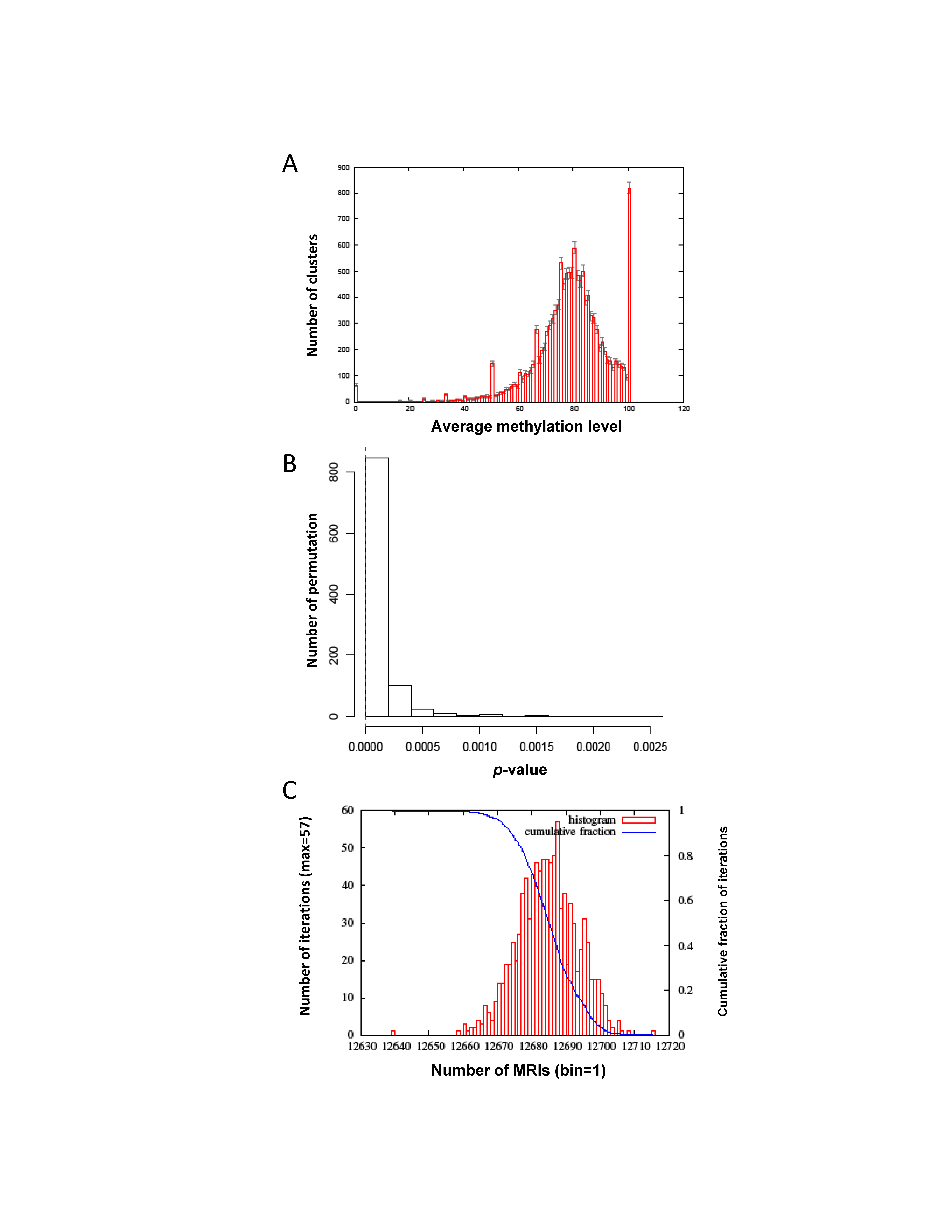

Supplement: Figure S3 — Results from a permutation test which examines whether the distribution of methylation levels of clusters was obtained by chance. A. Average methylation levels of clusters in 1000 permutation. The Y-axis represents the average number of MRIs for a bin of methylation levels and the error bars represent standard deviation. B. Histogram of p-values in 1000 permutation. The p-values were calculated by a student t-test between the original and permutated distributions. C. Histogram of MRIs in 1000 permutation. After discarding clusters of equal or less than 20% methylation, MRIs were determined in each permutation. The minimum, mean, and maximum numbers of MRIs are 12,369, 12,684, and 12,715. The distribution was far from 11,971 and demonstrated that the distribution was not obtained by chance. (2.05 MB TIF) [file pone.0013020.s003.tif]

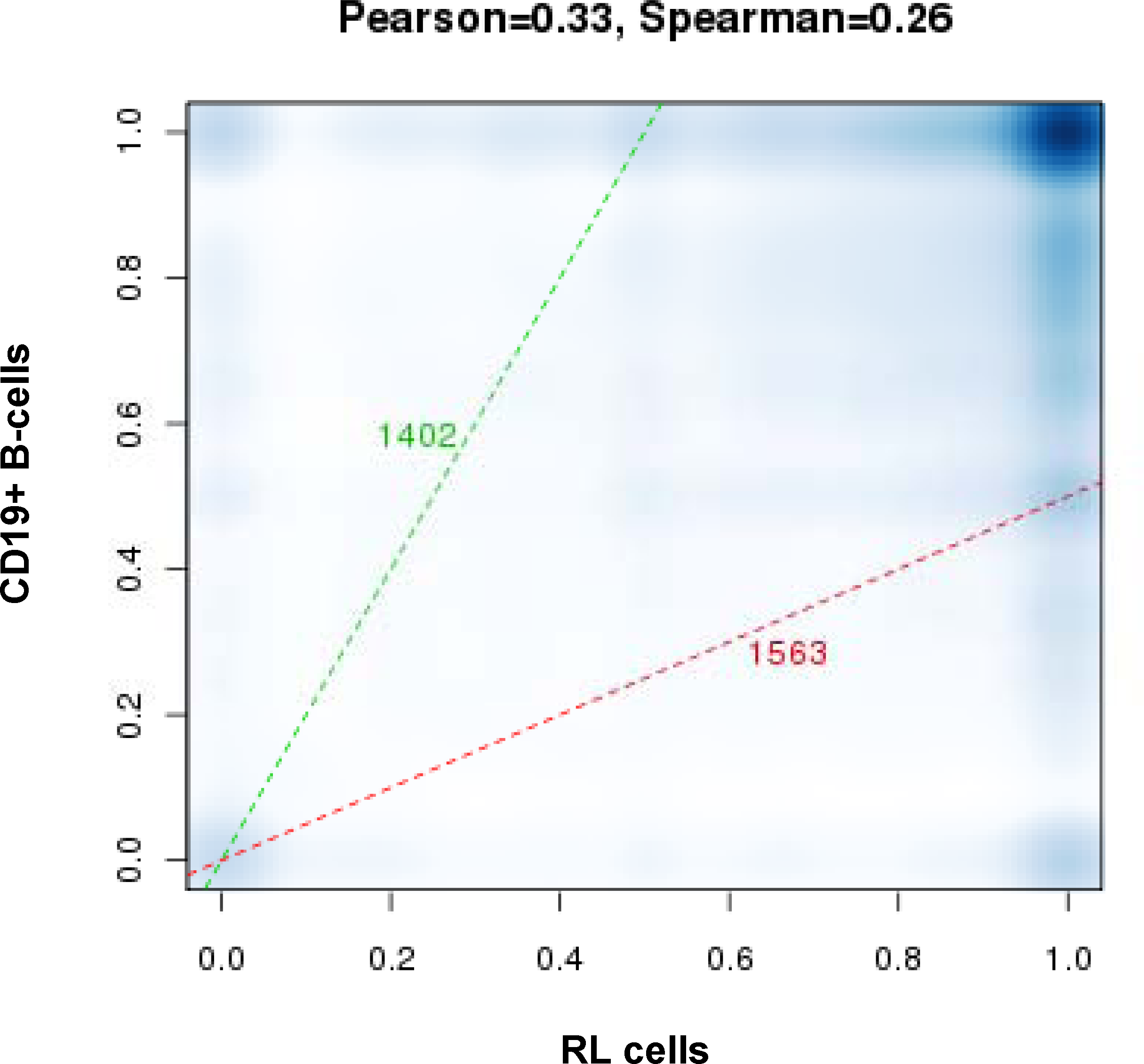

Supplement: Figure S4 — Comparison of the methylation levels of overlapping CpG sites between RL and CD19+ B-cells. A Pearson correlation coefficient of 0.33 was observed between the two samples, suggesting significantly differential methylation. (0.66 MB TIF) [file pone.0013020.s004.tif]

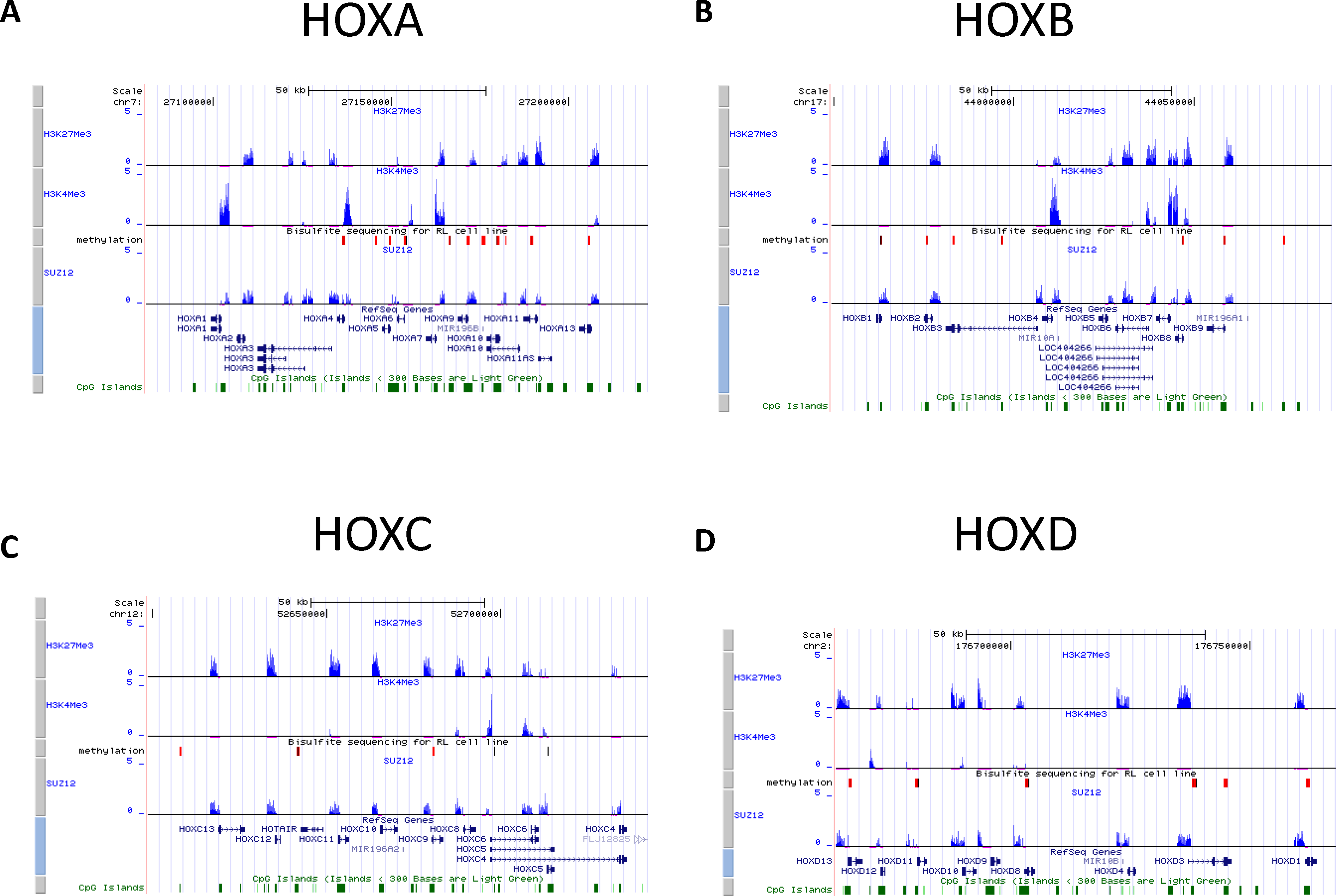

Supplement: Figure S5 — DNA methylation and histone modification profiles of 4 Hox gene clusters in RL cells. Each blue bar corresponds to the log2 ratio of ChIP/Input DNA for an individual probes. The methylation tracks are the same as described in the paper. (1.65 MB TIF) [file pone.0013020.s005.tif]

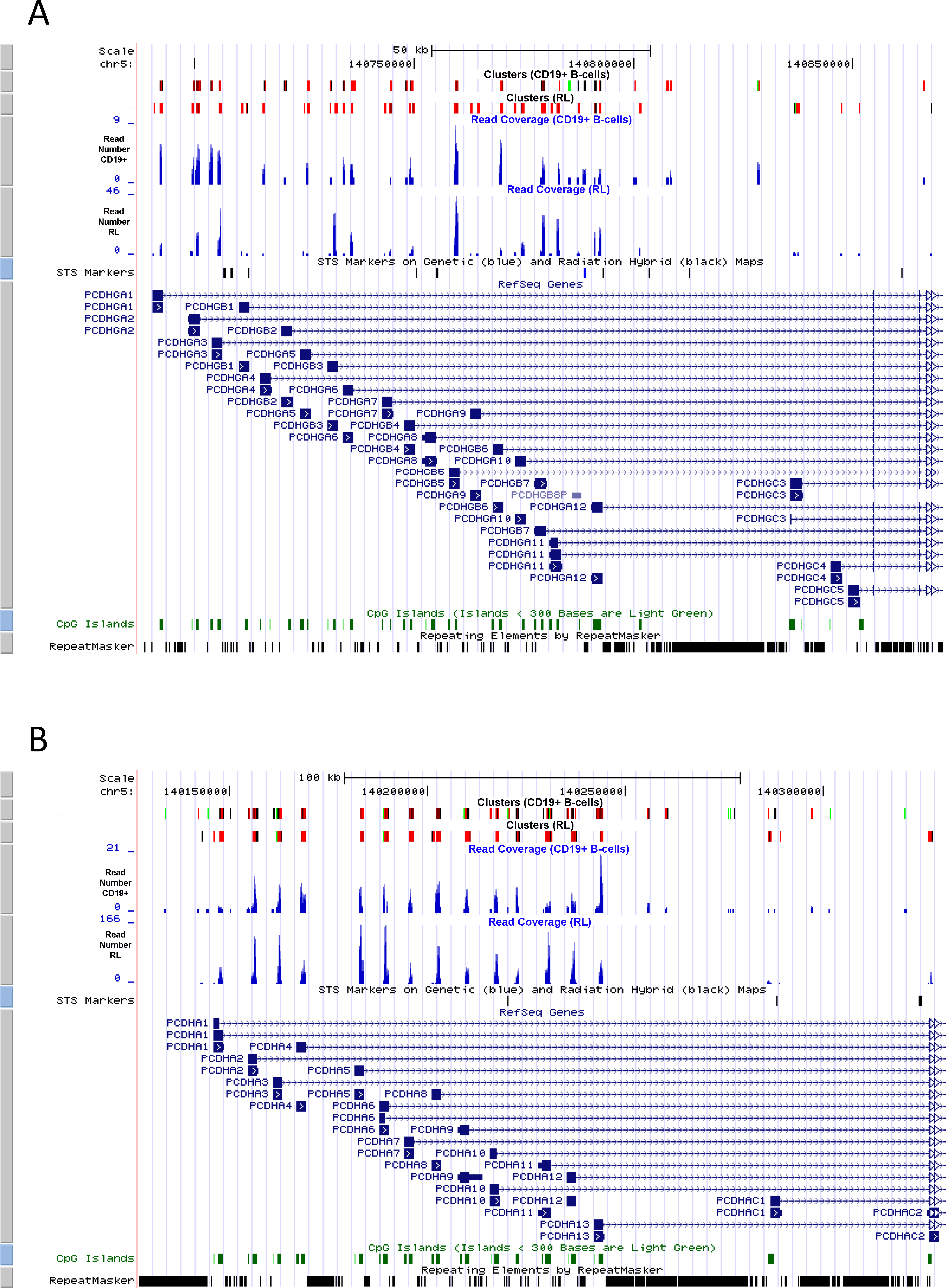

Supplement: Figure S6 — Bisulfite sequencing results of protocadherin gene clusters. A. Alignment results of bisulfite sequencing reads in the PCDHGA-B gene clusters. B. Alignment results of bisulfite sequencing reads in the PCDHA gene clusters. Custom tracks were uploaded into the UCSC genome browser. The vertical line in red color indicates the level of methylation at each CpG site. The deeper red shade indicates the higher methylation level. The blue color peaks show the sequencing coverage for each cluster. (3.26 MB TIF) [file pone.0013020.s006.tif]

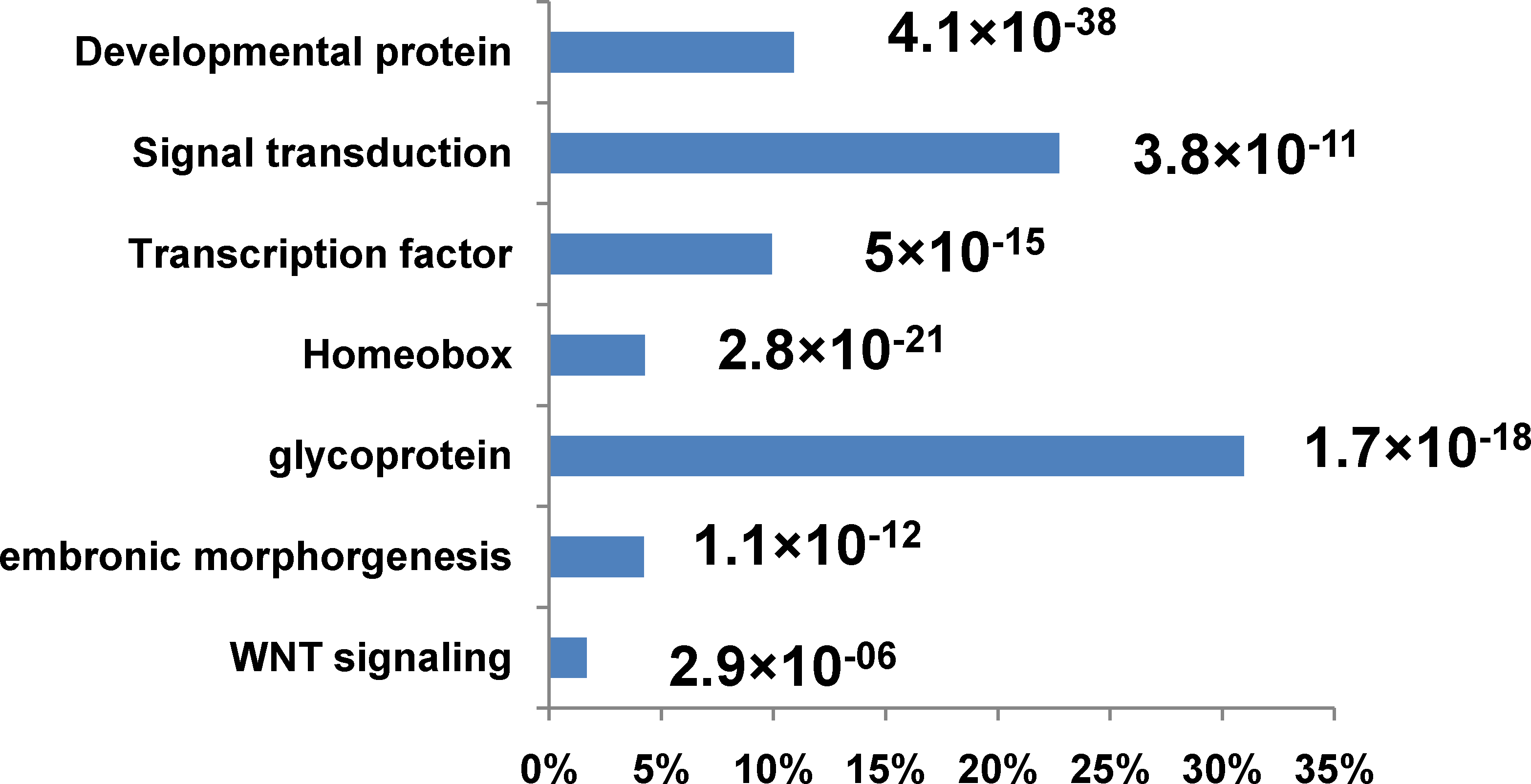

Supplement: Figure S7 — Functional analysis of genes associated with MRIs in the 5′-end. Enrichment scores from analysis of 1,817 annotated genes hypermethylated in RL using DAVID (http://david.abcc.ncifcrf.gov/). The x-axis shows the percentage of methylated genes that fall into functional groups. y-axis shows the functional group. The p-value represents the likelihood that a group of genes are NOT enriched by chance. (0.14 MB TIF) [file pone.0013020.s007.tif]

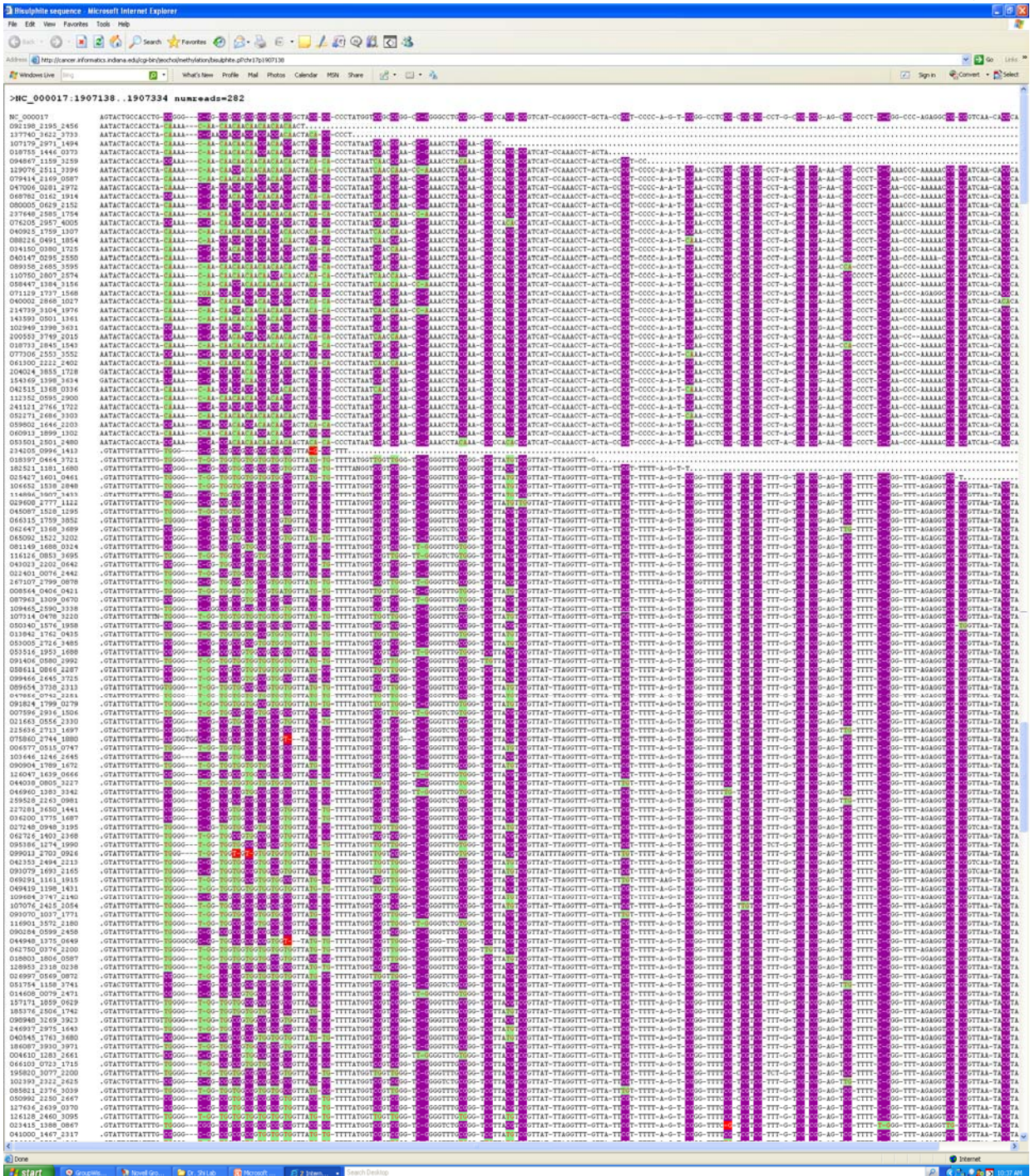

Supplement: Figure S8 — The HIC-1 gene, an example of bisulfite sequencing alignment results. The custom track on the top panel shows the methylation levels. Red color indicates the methylated CpGs. The lower panel shows the externally linked multiple alignment results. The methylated and unmethylated CpGs are also highlighted using different colors. (0.53 MB PDF) [file pone.0013020.s008.pdf]

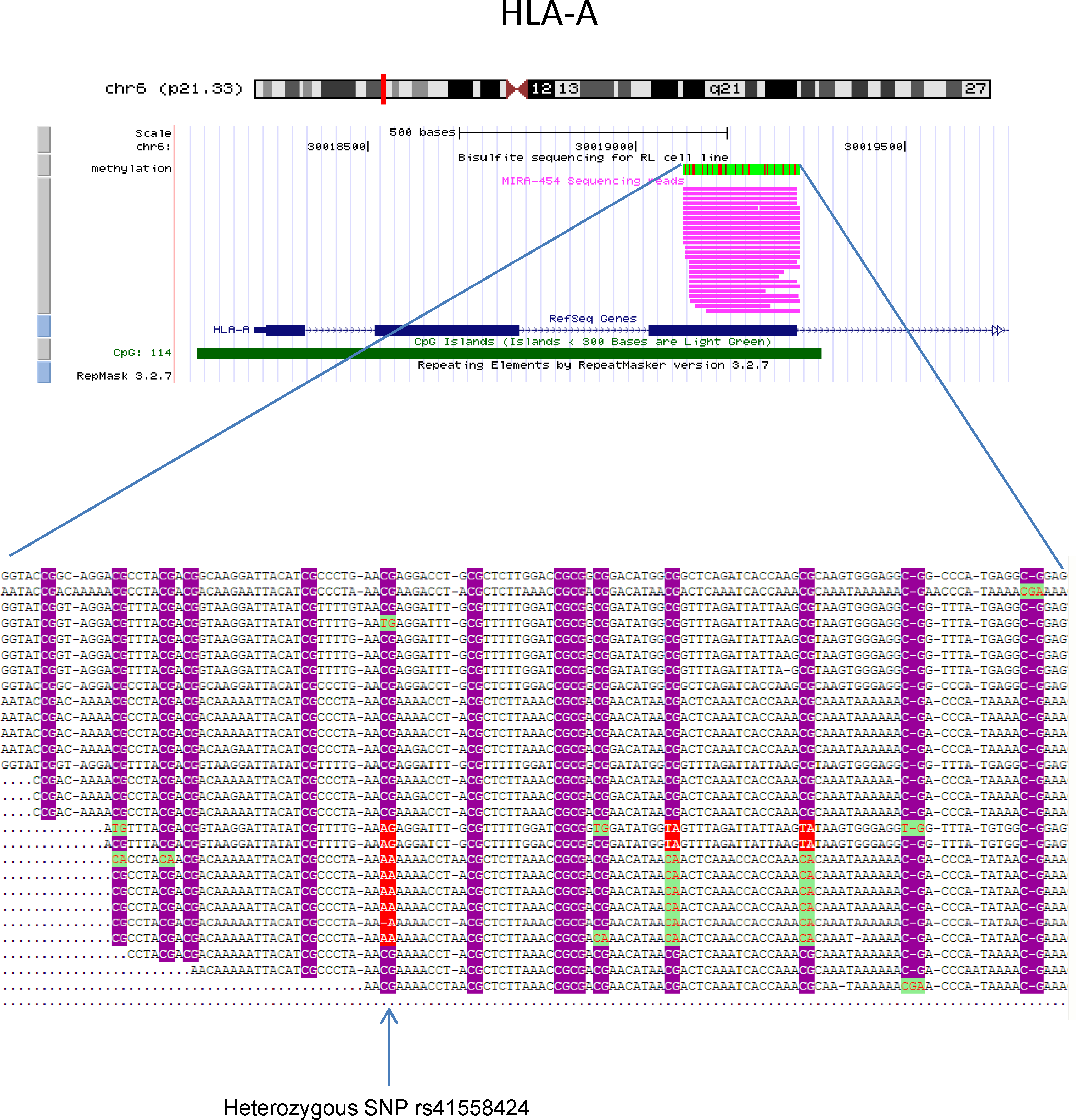

Supplement: Figure S9 — The HLA-A gene, another example of bisulfite sequencing alignment results. The custom track on the top panel shows the methylation levels. Red color indicates the methylated CpGs. The lower panel shows multiple alignment results. The methylated and unmethylated CpGs are also highlighted using different colors. A heterozygous polymorphism (rs41558424) seems to correlate with the two unmethylated CpG sites that are 30 to 50 bp downstream of the SNP. (4.15 MB TIF) [file pone.0013020.s009.tif]

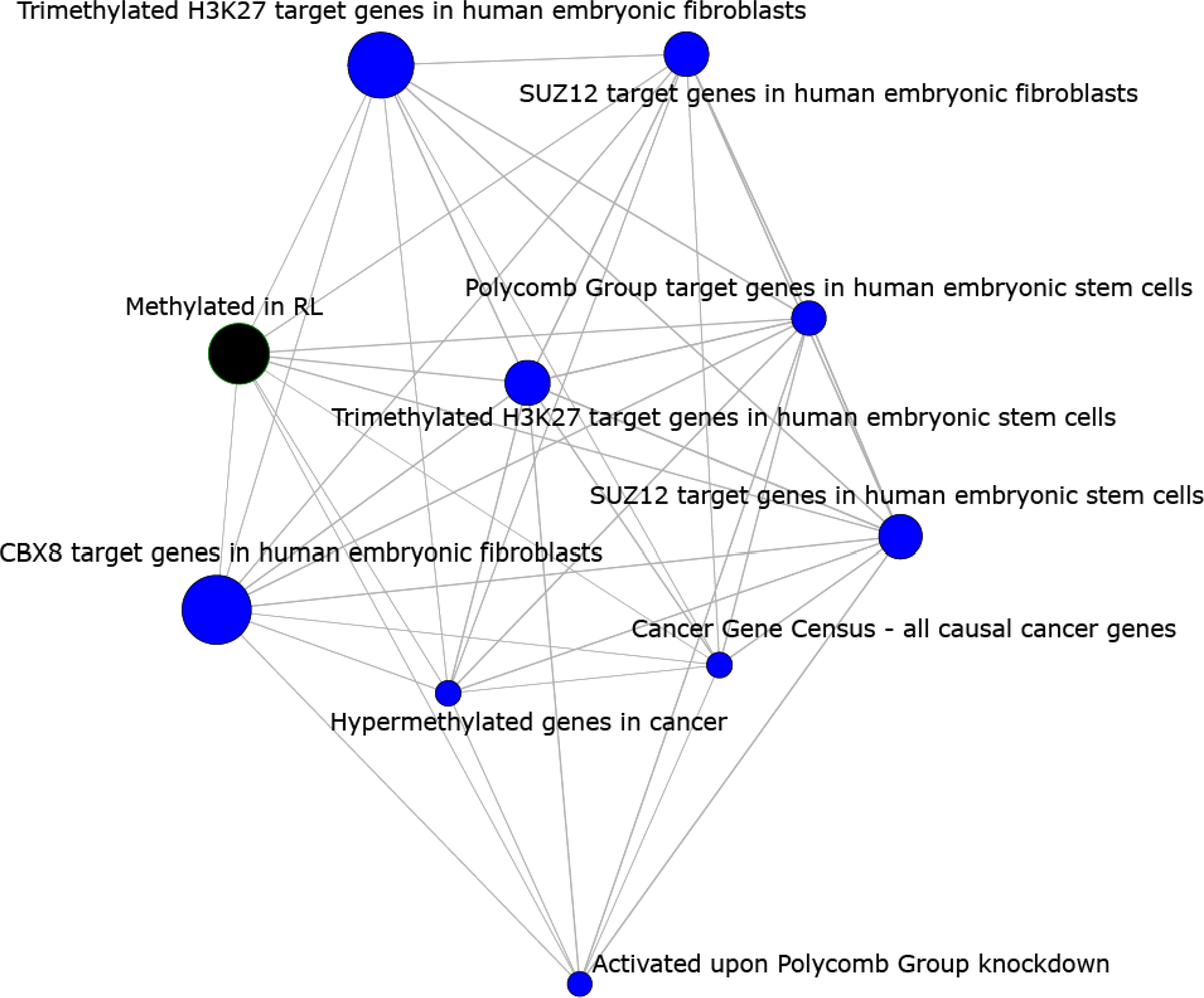

Supplement: Figure S10 — Oncomine concepts map of methylated genes in RL compared to the known polycomb target genes in ES cells. Node represent molecular concepts (biologically related gene sets. Node size is proportional to the number of genes in the concept. Each edge represents a significant enrichment (p<0.05). (1.26 MB TIF) [file pone.0013020.s010.tif]

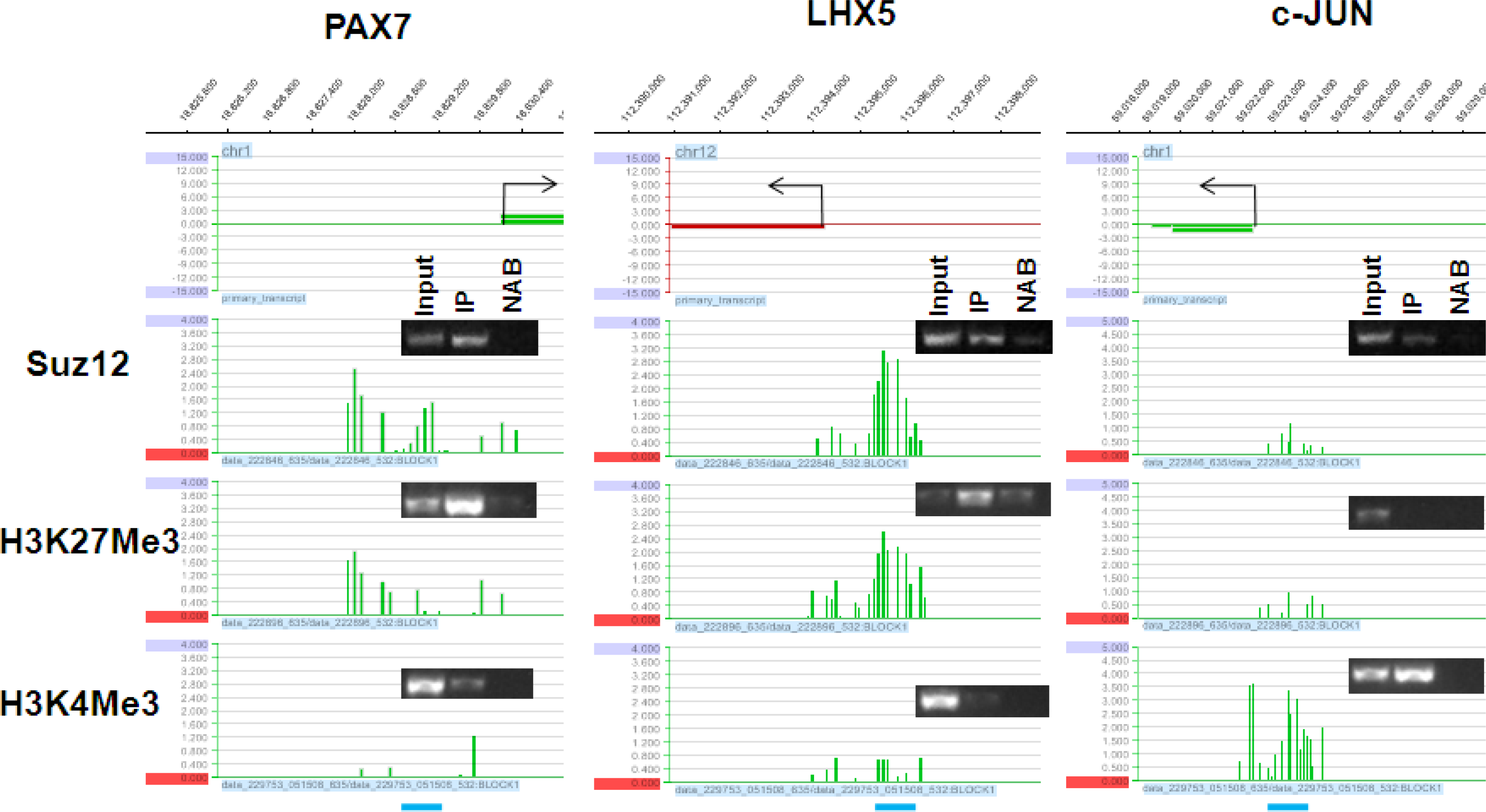

Supplement: Figure S11 — Mapping of Suz12, H3K4Me3 and H3K27Me3 to their target promoters. The NimbleGen promoter oligonucleotide array was hybridized with amplicons prepared from ChIP experiments using antibodies against SUZ12, H3K4Me3 and H3K27Me3, and the input control. Shown are three promoter regions: two that were repressed by SUZ12 (PAX7 and LHX5) and one that is not repressed by SUZ12 and actively transcribed (c-Jun). The fold enrichment was calculated by dividing the SUZ12 or H3K4Me3 and H3K27Me3 hybridization intensity signal by the input control signal for each oligonucleotide probe. Each green bar corresponds to the log2 ratio of ChIP/Input DNA for an individual probes. The inserts within each graph show independent ChIP confirmation using PCR analysis. The primers used in the PCR analysis were designed to span the region (blue horizontal bar) showing the highest peak of enrichment for each promoter. (0.85 MB TIF) [file pone.0013020.s011.tif]
